# History of the invasive African olive tree in Australia and Hawaii: evidence for sequential bottlenecks and hybridization with the Mediterranean olive

**DOI:** 10.1111/eva.12110

**Published:** 2013-11-28

**Authors:** Guillaume Besnard, Jérémy Dupuy, Maximilien Larter, Peter Cuneo, David Cooke, Lounes Chikhi

**Affiliations:** 1 Laboratoire Evolution & Diversité Biologique, CNRS, UPS, ENFA, UMR 5174Toulouse, France; 2 INRA, UMR 1202 BIOGECO, Université de BordeauxTalence, France; 3 The Australian Botanic Garden, Mount Annan, Royal Botanic Gardens and Domain TrustMount Annan, NSW, Australia; 4 Department of Primary Industries and Resources PIRSA, Biosecurity SAAdelaide, SA, Australia; 5 Instituto Gulbenkian de CiênciaOreiras, Portugal

**Keywords:** admixture, approximate Bayesian computation, biologic invasion, *cuspidata*, introgression, microsatellites, *Olea europaea*, plastid DNA

## Abstract

Humans have introduced plants and animals into new continents and islands with negative effects on local species. This has been the case of the olive that was introduced in Australia, New Zealand and Pacific islands where it became invasive. Two subspecies were introduced in Australia, and each successfully invaded a specific area: the African olive in New South Wales (NSW) and the Mediterranean olive in South Australia. Here, we examine their origins and spread and analyse a large sample of native and invasive accessions with chloroplast and nuclear microsatellites. African olive populations from the invaded range exhibit two South African chlorotypes hence supporting an introduction from South Africa, while populations from South Australia exhibit chlorotypes of Mediterranean cultivars. Congruently, nuclear markers support the occurrence of two lineages in Australia but demonstrate that admixture took place, attesting that they hybridized early after introduction. Furthermore, using an approximate Bayesian computation framework, we found strong support for the serial introduction of the African olive from South Africa to NSW and then from NSW to Hawaii. The taxon experienced successive bottlenecks that did not preclude invasion, meaning that rapid decisions need to be taken to avoid naturalization where it has not established a large population yet.

## Introduction

Biologic invasions are increasingly recognized as one of the major threats to biodiversity worldwide (Mooney and Cleland 2001; Clavero and García-Berthou 2005). This is particularly true on islands where recent invasions have led to the extinction of many endemic species (Blackburn et al. 2004; Sax and Gaines 2008). Invasive species can have dramatic effects through competition with or predation of native organisms and disturbance of ecosystem functioning (Wilcove et al. 1998; Davis et al. 2005; Richardson and Pysek 2006). Invasive populations are often thought to originate from a very limited number of individuals and therefore challenge the idea that populations going through bottlenecks should suffer from inbreeding and reduced fitness (Facon et al. 2011). To better understand the process of an invasion of a new territory, determining the origins of invasive species as well as the past and incipient evolutionary processes is essential. Several recent studies suggest that exotic species success is dependent on variable trait combinations, which makes it difficult to identify general determinants of invasiveness (Facon et al. 2006; Van Kleunen et al. 2010; Gurevitch et al. 2011). Specific studies are thus required to understand the recent evolutionary history of invasive species.

The olive tree (*Olea europaea* L., Oleaceae, hereafter ‘the olive’) is often associated with Mediterranean regions, but it is also known to be invasive and disruptive to the local flora, particularly in South Australia, New South Wales (NSW), Norfolk Island, northern New Zealand (e.g. Kermadec, Auckland Bay), the Hawaii archipelago and Saint Helena (Spennemann and Allen 2000; Cuneo and Leishman 2006; GISD 2010). Two olive subspecies have been spread by humans (Cuneo and Leishman 2006; Besnard et al. 2007a): *Olea europaea* subsp. *europaea* (the Mediterranean olive) and *O. e*. subsp. *cuspidata* (Wall. ex G. Don) Cif. (the African olive). The subspecies show a distinct native regional distribution (Green 2002; Besnard et al. 2012) – *O. e. europaea* is a characteristic taxon of the Mediterranean Basin, while *O. e. cuspidata* is distributed from southern and eastern Africa to southern Asia (Pakistan, India, Iran and China). The two subspecies are generally easy to distinguish based on morphological traits (Médail et al. 2001; Green 2002; Cuneo and Leishman 2006), and their long geographical isolation also led to a high, significant genetic divergence (Rubio de Casas et al. 2006; Besnard et al. 2007b). The Mediterranean olive was one of the first woody crops and was spread by human cultivation during the last six millennia (Kaniewski et al. 2012). Unlike the *europaea* subspecies, *O. e. cuspidata*'s fruit has no commercial value, but the African olive has been exploited for its hard and durable wood and can be used as a rootstock, ornamental or hedging plant (Spennemann and Allen 2000; Starr et al. 2003).

The history of both invasive olive subspecies is only partially documented (Dellow et al. 1987; Cuneo and Leishman 2006). The agricultural development of Australia gained momentum in the early 1800s and coincided with the introduction of many plants from Africa and the Mediterranean that were climatically suited to Australia. The Mediterranean olive tree was one of the earliest plant introductions into Australia by agricultural pioneer John Macarthur in 1805. Since then, multiple clones have been introduced, and more than 100 olive varieties are presently reported (Sweeney and Davies 1998). During the mid-1800s, the Macarthur family operated a large nursery at the famous Camden Park estate in south-west Sydney, NSW, and shipped potted plants throughout the colony. Plant listings in the 1843 Camden Park Nursery catalogue include a number of introduced plants that have since become environmental weeds, including African olive, which was established at this time. Isolated trees of African olive were also reported in the Adelaide region, South Australia (Shepherds Hill; Cuneo and Leishman 2006). In contrast to continental Australia, the origins of invasive olive in the oceanic islands and archipelagos are not clearly documented (GISD 2010). The infestation on Norfolk Island by the African olive is probably relatively old (during the 19th century; Cuneo and Leishman 2006), while the first records on Maui (Hawaii) and Saint Helena date back to the 1960s and early 2000s, respectively (Starr et al. 2003; GISD 2010).

While human activities contribute to transcontinental dispersal of *O. europaea*, birds are responsible for its local spread (Spennemann and Allen 2000). The dispersal range and the amount of seeds dispersed depend on the animal species but probably also on the size of the fruits (Alcantara and Rey 2003). After dispersal and establishment, the olives outcompete the native vegetation (such as eucalypts) by preventing regeneration. *Olea europaea* forms a crown under which olive seedlings can grow, but most native flora cannot (Cuneo and Leishman 2006; Cuneo et al. 2010; Major 2010). For example, the formation of African olive canopy in the Cumberland plain woodland resulted in a 78% reduction in native understory plant richness (Major 2010). In addition, the establishment of African olive can affect the local fauna by changing the vegetation structure and fruit availability. The speckled warbler has been shown to be negatively affected by the African olive invasion, while nonindigenous bird species such as the common starling and Eurasian blackbird are attracted by the presence of the African olives. This further encourages the displacement of the native fauna (DECC 2007). Not only can olive trees thrive in dry woodlands, they are also highly invasive in coastal regions. Hence, olives are considered as a serious threat to the biodiversity of Australia (Manders and Richardson 1992; Tozer 2003; Cuneo and Leishman 2006; GISD 2010).

The use of genetic data can be useful in reconstructing the history and hence identifying the source of invasions and documenting the population dynamics of invaders (Estoup et al. 2004; Bonhomme et al. 2008; Wilson et al. 2009; Ascunce et al. 2011; Lander et al. 2011; Ndlovu et al. 2013). Such information not only increases our understanding of the ecological constraints of the native habitat of the invader (by comparing the invasive and native habitats), but it can also help unravel evolutionary changes that have occurred since it was introduced (Prentis et al. 2008; Dlugosch and Parker 2008; Rey et al. 2012). Previous genetic characterizations of invasive *Olea*, using both plastid DNA and nuclear markers, have located the potential geographical origins of these invasive populations (Besnard et al. 2007a). Populations near Adelaide (subsp. *europaea*) showed high genetic similarities with central and western Mediterranean cultivars, while Hawaii and NSW populations (subsp. *cuspidata*) showed a genetic affinity with southern African populations. An event of early admixture between *europaea* and *cuspidata* subspecies was reported (Besnard et al. 2007a), indicating that hybridization could have played a role in the invasion of the two olive taxa. Yet, this hypothesis was strongly criticized by other authors (Breton et al. 2008), who argued that these two subspecies are not in contact in the invasive range. Furthermore, Besnard et al. (2007a) found that the NSW *cuspidata* population displayed reduced genetic diversity compared with a native population from South Africa, suggesting a strong bottleneck during the introduction in Australia. The genetic diversity found in the Hawaiian population was even lower than in NSW. The hypothesis of sequential introductions was stated but still needs to be tested. Under this scenario, the first introductions may have occurred from southern Africa to NSW, and then, NSW may have been a source of invaders for other regions such as the Hawaii archipelago (Spennemann and Allen 2000; Starr et al. 2003; Besnard et al. 2007a).

Recent advances in population genetics have generated methods to reconstruct the past demographic history of species. Coalescent theory and Bayesian analysis have provided a major framework that led to the development of several inferential methods to study changes in population size (Hudson 1990; Beaumont 1999). In many cases, the methods were computationally very demanding even for simple models (full-likelihood methods). More recently, an alternative framework, called approximate Bayesian computation (ABC; Beaumont et al. 2002), has emerged. This flexible framework has been particularly successful for the estimation of population parameters under complex demographic histories, especially to investigate the recent colonization history of invasive species (Pascual et al. 2007; Beaumont 2010; Csilléry et al. 2010; Estoup and Guillemaud 2010; Estoup et al. 2010; Lombaert et al. 2011; Sousa et al. 2012).

The purpose of our study was to examine the origins and spread of invasive olives in Australia and Hawaii using both plastid and nuclear markers. A large sample of invasive and native accessions was characterized and provided strong evidence for the Mediterranean (*europaea*) and African (*cuspidata*) origins of Australian invasive olives as well as for putative admixture between the subspecies. Then, we used an ABC framework to identify the most probable among different colonization scenarios and to infer several key parameters of the foundation history of olives in East Australia and Hawaii (e.g. duration of bottlenecks, effective number of founders). Because of the multiple introductions of Mediterranean cultivars (clones) from various geographic origins to Australia, probably in numerous sites over the last 200 years, it appears difficult at this stage to model the complex origin of invasive European olive. The presented ABC analyses were thus only applied to reconstruct the demographic history of the invasive African olive in Australia and Hawaii, which seems to be much simpler, based on the results from the previous and present studies.

## Material and methods

### Plant sampling

We previously showed that invasive olive populations from South Australia and NSW have probably derived from introductions of Mediterranean and southern African trees (Besnard et al. 2007a,b2007b). To better document the geographic origins of invasive populations, we used both plastid and nuclear DNA markers to characterize native and invasive trees of both subspecies *cuspidata* and *europaea*. For each marker, we used a different sample of trees.

**Figure 1:**
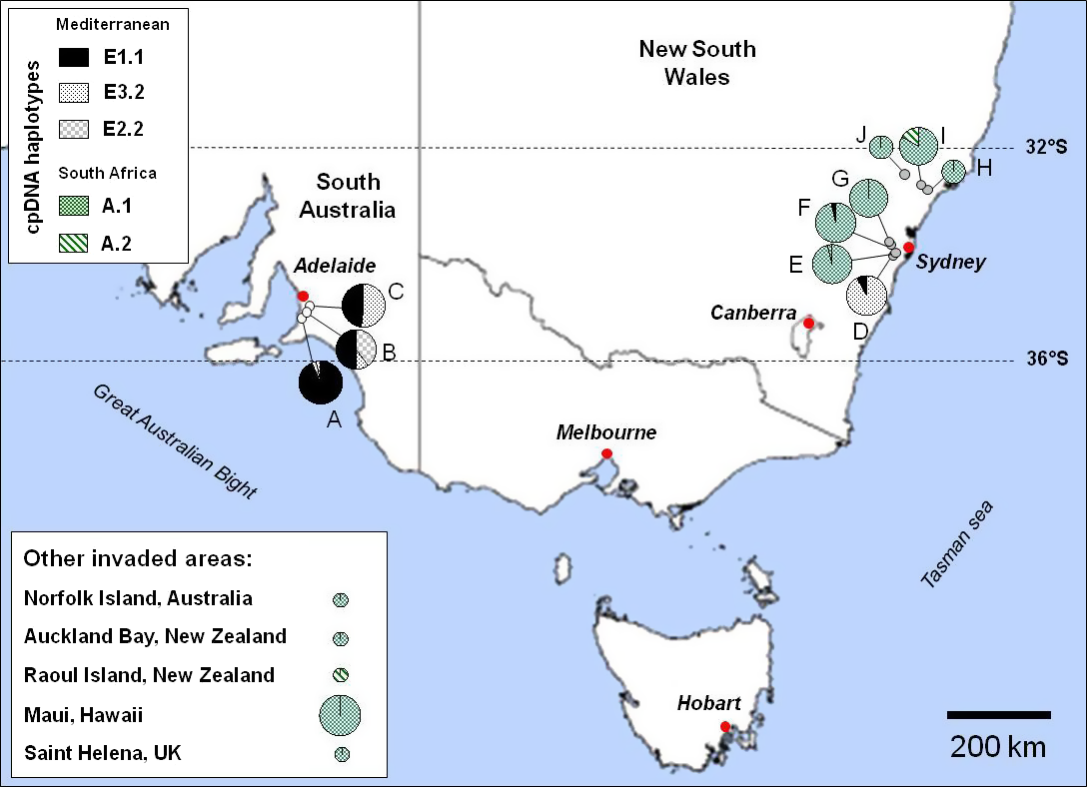
Geographical locations of the ten populations sampled in Australia and detected chlorotypes form the whole invasive range. A = Lonsdale, B = Shepherds Hill, C = Brownhill Creek, D = Camden Park, E = Mount Annan, F = Bringelly, G = Luddenham, H = Maitland Park, I = Harpers Hill and J = Ravensworth. The frequency of chlorotypes found at each location is indicated. Size of pie charts is proportional to the number of individuals analysed.

First, the plastid DNA (cpDNA) variation was investigated on a large sample of trees (2126 individuals). A recent study reported cpDNA haplotype profiles for 1797 trees of subsp. *europaea* (including 534 Mediterranean cultivars and 1263 oleasters; Besnard et al. 2013). Here, we characterized 81 accessions from 30 locations covering the whole native range of the African olive (Table S1) and representative of lineages A, C1 and C2 (Besnard et al. 2007b). In addition, 244 individuals from 11 locations were sampled in the invasive olive range (Table S1): ten locations in Australia (NSW locations: Bringelly, Luddenham, Mount Annan, Camden Park, Harpers Hill, Ravensworth, Maitland Park; South Australia locations: Shepherds Hill, Lonsdale, Brownhill Creek; Fig. 1) and one location in Hawaii (Maui). Lastly, to test for putative multiple origins of populations of African olive in its whole invasive range (GISD 2010), four additional herbarium *cuspidata* samples from Raoul Island, Auckland Bay, Norfolk Island and Saint Helena Island were also characterized (Table S1).

Second, to investigate the colonization history of invasive populations, we analysed a subsample of 332 trees with nuclear microsatellites (see below). The 11 invasive populations from Australia (218 individuals) and Hawaii (26 individuals) were characterized and compared with putative source gene pools from the native range, namely the Cape Town location for the African olive (20 individuals), and a set of Mediterranean cultivars for subsp. *europaea*. The 68 Mediterranean cultivated accessions are listed in Table S2. They were chosen to represent cultivars from the East, Central and West Mediterranean gene pools (Haouane et al. 2012). Four invasive locations (i.e. Bringelly, Brownhill Creek, Lonsdale and Maui) were partially characterized with eight nuclear microsatellite markers in a previous study (Besnard et al. 2007a).

In continental Australia, the three main areas invaded by olive trees – Adelaide hills (sites A-C; South Australia), Cumberland plain (sites D-G; NSW) and Central Hunter region (sites H-J; NSW) – were thus sampled (Cuneo and Leishman 2006) and analysed with both plastid and nuclear markers. Among locations of Cumberland plain, Camden Park is considered as an initial introduction site for cultivated olives (subsp. *europaea*) in eastern Australia during the early 18th century (Dellow et al. 1987; P. Cuneo, personal observation), and there, the Mediterranean taxon was probably in contact with subsp. *cuspidata*. Trees from this park are unusually big, and many trees are considered to be more than 100 years old; most of them (22/25) were male sterile (P. Cuneo, personal observation; observations performed in November 2010), producing nondehiscent pollen with of mix of tetrads and aborted microspores [reported as phenotype *ms2* by Besnard et al. (2000)].

### Genetic characterizations

Genomic DNA of each individual was extracted from c. 20 mg of silica-dried leaf using the Plant-DNeasy Minikit (QIAGEN Inc., GmbH, Hilden, Germany). The plastid genome is maternally inherited in olive, and strong geographic patterns of cpDNA variation have been observed making it very useful for identifying the origins of introduced material (Besnard et al. 2000, 2011). For an optimal identification of cpDNA haplotypes (or chlorotypes) among all sampled accessions, 64 cpDNA loci (i.e. microsatellites, indels and single-nucleotide substitutions) were first investigated as reported by Besnard et al. (2011). The genetic diversity within invasive populations and putative source gene pools was then investigated with eleven nuclear microsatellite loci [or simple sequence repeats (SSRs)]: ssrOeUA–DCA1, ssrOeUA–DCA3, ssrOeUA–DCA5, ssrOeUA–DCA8, ssrOeUA–DCA9, ssrOeUA–DCA14, ssrOeUA–DCA15, ssrOeUA–DCA18 (Sefc et al. 2000), EMO03 (de la Rosa et al. 2002), GAPU71A (Carriero et al. 2002) and PA(ATT)2 (Saumitou-Laprade et al. 2000). These loci were chosen for their high-to-moderate polymorphism level (e.g. *H*_*T*_ ranging from 0.40 to 0.95 in native populations) and a low frequency of null alleles in previous studies (Besnard et al. 2007a). For each locus, the forward primer was labelled with a fluorochrome. Two types of fluorochromes were used: HEX (green) and FAM (blue). Amplification of each locus was performed separately using previously described procedures (Baali-Cherif and Besnard 2005). Three-locus multiplexes were prepared [i.e. PA(ATT)2/DCA08/DCA09/DCA01/DCA03, DCA18/DCA05/GAPUI71A and DCA14/EMO03/DCA15] to which 0.02 μl of ROX-500 (Applied Biosystems, Foster City, CA, USA) was added as a reference for the size of DNA segments. Electrophoresis of PCR products was performed using an ABI PRISM® 3100 Genetic Analyzer (Applied Biosystems), and allele size of each locus was then determined with GeneMapper.

### Data analyses

#### Plastid haplotype networks

A plastid DNA profile (chlorotype) was defined for each African olive accession by the combination of alleles from all plastid loci. Based on these data, the relationships among chlorotypes were visualized by constructing a reduced median network implemented in Network version 4.112 (Bandelt et al. 1999). Multistate microsatellites were treated as ordered alleles and coded by the number of repeated motifs for each allele (e.g. number of T or A), whereas the presence or absence of other indels was coded as 1 and 0, respectively. Basically, this coding strategy assumes that variation at cpDNA microsatellites is mainly due to single-step mutations (e.g. Besnard et al. 2007b), while allowing consideration of length polymorphisms (microsatellites or indels) with similar weight. The analysis was performed on the whole *cuspidata* data set and then only on sub-Saharan chlorotypes (lineage A).

For the Mediterranean subspecies, we only detected chlorotypes already reported in the cultivated olive (see below) by Besnard et al. (2013), who reconstructed the whole haplotype network for that subspecies. We thus refer readers to this study.

#### Population genetic analyses

First, parameters of genetic diversity in the investigated populations or gene pools (i.e. cultivars) were estimated. The observed heterozygosity (*H*_O_), the expected heterozygosity (*H*_S_) and *F*_IS_ were calculated from allele frequencies at each nuclear SSR locus using Fstat version 2.9.4 (Goudet 2005). Significance of *F*_IS_ was tested by estimating the 95% confidence interval of the value for each population using the bootstrap approach implemented in Genetix (Belkhir et al. 2004). To account for the difference in sample sizes, allelic richness (*R*_S_) rather than the number of alleles was also estimated using Fstat. A Wilcoxon paired test (two-sided) was then used to evaluate the significance of differences among genetic diversity measures between invasive and native populations. Pairwise *F*_ST_ was also computed using Fstat between the ten invasive populations. Significance of pairwise differentiation was assessed using Bonferroni corrections. Relative contribution of gene flow by pollen versus seeds (*r*) was estimated according to Ennos (1994), but only in South Australia where substantial plastid DNA diversity was observed (*H*_T_ = 0.50; see results) allowing estimating *F*_ST_ values for cytoplasmic markers.

Structure version 2.3.4 (Pritchard et al. 2000) was used to estimate the number of genetic clusters in our data set. This model-based clustering method uses multilocus genotype data to infer population structure and assign individuals to populations. Using the *ad hoc* Δ*K* statistic based on the rate of change in the log probability of data between successive *K* values, Structure can be used to identify the number of genetic clusters (Evanno et al. 2005). For each *K* value that was retained, each accession was assigned to each cluster with a posterior membership coefficient (*p*).

#### Demographic models and introduction scenarios

An ABC approach was used to infer the recent colonization history of invasive African olive in Australia and Hawaii. In this study, we show that invasive trees of subsp. *cuspidata* found in Australia and Hawaii were most probably introduced from South Africa or at least belong to the same genetic cluster (see below). We thus focused on this taxon to determine which among several scenarios of sequential colonization was the most probable. Only nuclear SSR data were used for these inferences because plastid DNA loci provide low variation among populations, especially in the invasive range (see below). We used two different implementations but, due to space limits, only the implementation based on the DiyABC version 2.0 program (Cornuet et al. submitted) is presented in detail here. The other implementation uses the *ms* program (Hudson 2002), which allows users to simulate extremely complex demographic histories, together with several in-house scripts and published R packages (see Data S1 for details). Three demographic scenarios or models were considered differing in the order of introductions all from a South African source (Fig. S1). In scenario 1, we assume a first introduction to Australia followed by a second introduction from Australia to Hawaii. In scenario 2, we assume a first introduction to Hawaii that is followed by second introduction from Hawaii to Australia. Finally, in scenario 3, we assume two independent introductions to Australia and Hawaii, which may have been at different times. For each scenario, several demographic parameters were defined: the current effective population size (in units of diploid individuals, not genes) for the three sampled populations (N_e1_, N_e2_ and N_e3_ for South Africa, Australia and Hawaii, respectively), the number of founders in the introduced populations (N_1_ and N_2_, respectively, for the first and second colonization events) and the duration of the initial bottleneck (db_i_) which may be seen as a latency phase assumed to have taken place just after the introduction events for the two invasive populations (db_1_ and db_2_, respectively). We note here that DiyABC only allows instantaneous population size changes (i.e. no linear or exponential increase or decrease). As a consequence, it is necessary to assume this latency phase to discretize a population size increase that may have been more gradual. Note also, that some authors (e.g. Crooks and Soule 1999; Facon et al. 2006) have suggested that invasive species have an initial ‘latency’ period during which the population size remains relatively constant and which is followed by a rapid growth. Finally, two other parameters correspond to the two introduction times, or split, for the two colonization events (respectively, T_1_ and T_2_). Because the intensity of a bottleneck event depends on both the number of effective individuals during this event and the duration of the event, we also considered a parameter combining two of the parameters already mentioned, N_i_ and db_i_. This parameter, K_i_ = N_i_/db_i_ was introduced by Wright et al. (2005) as a measure of the bottleneck severity, and it was separately estimated for Australian and Hawaiian populations (K_1_ and K_2_, respectively). We note, however, that K is inversely related to the severity of the bottleneck and that small values correspond to severe bottlenecks and large values to less severe bottlenecks.

For all these demographic parameters, prior distributions have been implemented according to the current knowledge on invasive olive (Table 1). We used, for all parameters, uniform distributions, represented by U[min, max], where min and max are the lower and upper bounds of the distribution. The prior distributions for N_e1_, N_e2_ and N_e3_ were set to U[10^4^, 10^5^] considering that the size of populations is large in the studied areas (Starr et al. 2003; Cuneo et al. 2009). For N_1_ and N_2_, we used U[2, 50] as suggested by the putative strong bottleneck during the olive introductions (Besnard et al. 2007a). For the split parameters (T_1_ and T_2_), and for db_1_ and db_2,_ we used values between one and 40 generations (i.e. U[1, 40], but see below for nonindependence issues) on the basis of historical knowledge about the presence of African olive in Australia and Hawaii (i.e. introduction during the last 200 years; Cuneo and Leishman 2006). We avoided the difficulty of estimating the generation time of this tree species by implementing a wide prior distribution. With an introduction event in Australia estimated at the beginning of 19th century, this prior encompasses short and long generation times (i.e. from 5 to 200 years/generation). Note that T_1_, T_2_, db_1_ and db_2_ are not independent as the order of the colonization imposes a constraint on T_2_ (T_2_ < T_1_), whereas T_1_ can take any value within the uniform prior defined above. Similarly, the latency periods cannot be longer than the colonization times (i.e. T_1_ > db_1_ and T_2_ > db_2_).

**Table 1:** Mean, median, mode and quantiles for demographic parameters and mutation rate under scenario 1. These results were obtained with DiyABC version 2.0 (Cornuet et al. submitted). Q_2.5%_ and Q_97.5%_ are the 2.5% and 97.5% quantile values, respectively. All values were estimated from 500 000 and 5000 simulated data for priors and posteriors, respectively. Modes were not given for N_e1_, N_e2_, N_e3_, N_1_, N_2_ and mu because their prior distributions were uniform.

Parameter	Mean	Median	Mode	Q_2.5%_	Q_97.5%_
N_e1_					
Prior	54 994	55 017	–	12 248	97 754
Posterior	18 390	16 380	11 857	10 256	37 591
N_e2_					
Prior	54 988	54 963	–	12 255	97 778
Posterior	59 870	57 880	83 290	12 728	98 262
N_e3_					
Prior	54 985	55 007	–	12 230	97 771
Posterior	51 500	49 470	18 751	11 634	97 549
N_1_					
Prior	26	26	–	3	49
Posterior	24.6	23.9	23.7	4.93	47.05
N_2_					
Prior	26	26	–	3	49
Posterior	12.1	9.8	5.26	2.38	36.76
db_1_					
Prior	16	16	4	1	35
Posterior	7.7	6.9	5.53	1.43	19.9
db_2_					
Prior	11	9	1	1	29
Posterior	13.8	13.3	11.96	3.1	27.4
K_1_					
Prior	3.45	1.52	0.99	0.15	21.5
Posterior	3.55	3.33	3.10	1.54	6.59
K_2_					
Priors	5.37	2.58	1.01	0.23	33
Posterior	0.98	0.77	0.59	0.34	2.46
T_1_					
Prior	33	34	40	17	40
Posterior	35.5	36.6	39.2	23.9	40
T_2_					
Prior	22	22	23	5	37
Posterior	20.2	20	19.8	7.7	33.1
mu					
Prior	5e-04	5.5e-04	–	1.2e-04	9.8e-04
Posterior	3.8e-04	3.6e-04	3.2e-04	1.3e-04	7.5e-04

N_e1_, N_e2_ and N_e3_ = population effective sizes (number of individuals) for South African, NSW and Hawaiian populations, respectively; N_1_ and N_2_ = number of founders for the first and second colonization events (in NSW and then Hawaii); db_1_ and db_2_ = duration of the initial bottleneck after introduction in NSW and Hawaii (number of generations), respectively; K_1_ and K_2_ = intensity of the bottleneck in NSW and Hawaii, respectively; T_1_ and T_2_ = introduction times for the two colonization events (number of generations); mu = SSR mutation rate per locus and by geneation.

We assumed that the SSRs evolved according to the stepwise mutation model (SMM) with a uniform mutation rate prior (μ) bounded between 10^−4^ and 10^−3^ for all loci. In agreement with the Structure analysis (see Results), NSW sampled populations were pooled and analysed as a unique population with the exception of 29 individuals that were identified as early-generation hybrids on the basis of the Structure inference (see below). Nuclear SSR diversity within and between populations was summarized with 12 different summary statistics. For the three populations, we computed nine within-population statistics [mean number of alleles (*A*), mean allele size variance (*V*) and mean heterozygosity across loci (*H*_T_; Nei 1987) for each population] and three between-population statistics, namely pairwise *F*_ST_ values (Weir and Cockerham 1984). All these statistics were computed using DiyABC.

To identify the most probable model, we computed the posterior probability of the three scenarios above using a logistic regression approach on the first 1% simulations (Fagundes et al. 2007; Beaumont 2008). We then estimated the posterior parameters for that model using the local linear regressions method of Beaumont et al. (2002). A *logit* transformation was applied to ensure that the estimated values were comprised within the prior limits (Cornuet et al. 2008).

To validate the results of our ABC modelling approach, we first simulated data under each of the three models and applied the ABC algorithm to these data sets to determine whether we would correctly identify the scenario under which they were simulated. To do this, we used the ‘confidence in model choice’ function, implemented in DiyABC, which allowed us to estimate the proportion of type-1 and type-2 errors. We also evaluated the sensitivity of our inferences by testing different priors.

Finally, we evaluated a Bayesian equivalent of goodness of fit of the selected scenario, using the model checking option of DiyABC version 2.0 (Cornuet et al. submitted). This option allows us to evaluate to what extent the selected scenario and associated posterior distributions are corroborated by the observed data. Briefly, if a model-posterior combination fits the observed data correctly, then data simulated under this combination with parameters drawn from posterior distributions should be close to the observed data. In order for the model fit to be considered good, the observed statistics had to fall within the distributions of simulated statistics. We here simulated 1000 data sets from the posterior distribution of parameters obtained under scenario 1 to estimate such distributions. Principal component analysis (PCA) applied on test statistics vectors was also used as a mean to visualize the fit between simulated and observed data sets. As recommended by Cornuet et al. (2010), we used summary statistics as test statistics that were not used for model selection or parameter estimation in previous ABC treatments (Table S11).

## Results

### Maternal origins of invasive olives

**Figure 2:**
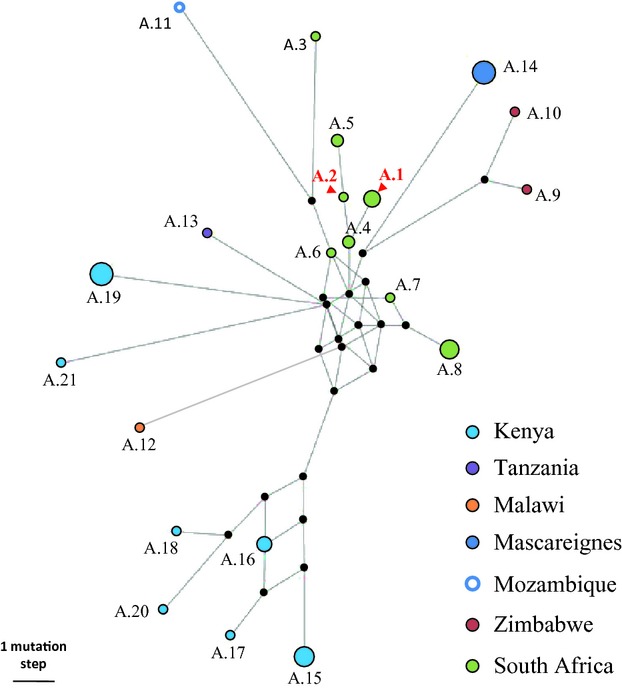
Reduced median network (Bandelt et al. 1999) of the 21 chlorotypes detected in the African range of subsp. *cuspidata* (lineage A; Besnard et al. 2007b). Each chlorotype is represented by a dot, whose width is proportional to the number of occurrences in our sample. See Table S3 for chlorotype profiles and geographic origins. The length of branches is proportional to the number of mutational steps. The missing, intermediate nodes are indicated by small black points. For each chlorotype, the country of origin is indicated by a specific colour. The two African chlorotypes detected in NSW are indicated in red (A.1 and A.2; both were detected in the population of Cape Town, South Africa).

In the Adelaide area (South Australia), we found three Mediterranean chlorotypes (namely E1.1, E2.2 and E3.2), while in the Sydney area (NSW), a mix of Mediterranean (E1.1 and E3.2) and African chlorotypes (A.1 and A.2) were detected as shown in Fig. 1 (see Table S3 for the profile of each chlorotype). The three Mediterranean chlorotypes have been previously reported in olive cultivars, although E2.2 is not frequent (approximately 1%) in the cultivated pool (Besnard et al. 2011, 2013). Chlorotypes A.1 and A.2 were also detected in the native range but only in the Cape region, South Africa (Table S3). These two chlorotypes are closely related (Fig. 2 and Fig. S2). In our invasive olive sample, they co-occurred only in the Harpers Hill population. Interestingly, the four herbarium African olive samples from northern New Zealand, Norfolk and Saint Helena also showed the same chlorotypes A.1 and A.2 (Fig. 1). Populations from the Cumberland Plain mainly harbour chlorotype A.1, except individual Bringelly no 21 (E1.1), Mt Annan no 17 (E3.2) and all Camden Park individuals which exhibit Mediterranean chlorotypes E1.1 or E3.2. Twenty-three of 25 individuals sampled in Camden Park displayed E3.2, and an examination of mature flowers indicates a male sterility phenotype *ms2* for all of them, but one which was not flowering, as previously reported in olive cultivars exhibiting E3 chlorotypes (formerly CCK; Besnard et al. 2000).

### Genetic structure of invasive populations based on nuclear SSRs and plastid DNA lineages

**Figure 3:**
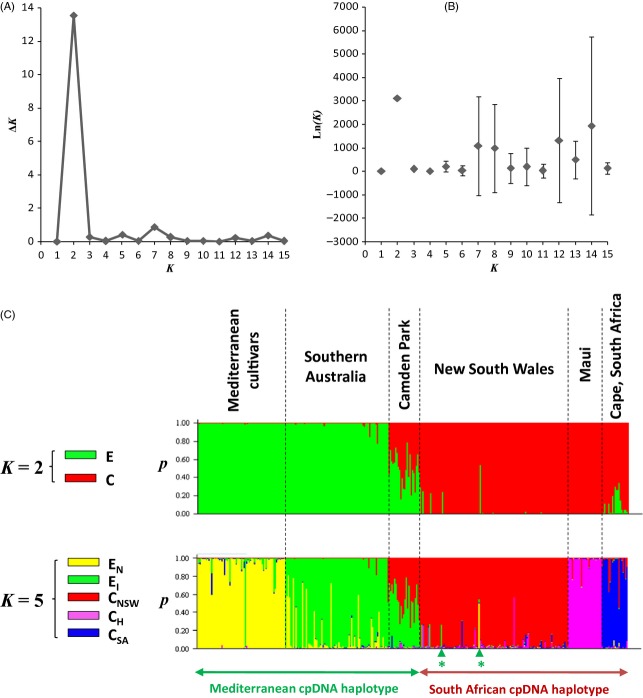
Inference of population structure in native and invasive olive accessions based on 11 nuclear SSRs and using Bayesian simulations with Structure (Pritchard et al. 2000). (A) Absolute values of the second-order rate of change of the likelihood distribution divided by the s.d. of the likelihoods (Δ*K*) for each *K* value; (B) Mean log likelihood [Ln(*K*) ± SD] averaged over the ten iterations for each *K* value; (C) Barplot of the Structure analysis based on the best two *K* values (i.e. 2 and 5) according to Ln(*K*) and Δ*K* criteria (Evanno et al. 2005). The percentage of assignment of each individual to the clusters averaged over ten iterations is shown. Each vertical bar represents an individual. The chloroplast lineages match with the two clusters defined on nuclear SSRs except for admixed individuals (*indicate Mt Annan no 17 and Bringelly no 21). At *K* = 2, clusters E and C reflect the strong genetic differentiation between subspecies *europaea* and *cuspidata*, respectively. At *K* = 5, native and invasive Mediterranean olives are mostly assigned to clusters E_N_ and E_I_, respectively. Similarly, African olive individuals from South Africa, NSW and Hawaii are mostly assigned to three distinct clusters, namely C_SA_, C_NSW_ and C_H_.

Based on the complete nuclear SSR data set (Table S4), two main clusters (E and C) were recognized in the Structure analysis (Fig. 3): the first includes all Mediterranean cultivars and the South Australian individuals (Lonsdale, Shepherds Hill and Brownhill Creek; subsp. *europaea*), while the second corresponds to native South African olive trees, most individuals from NSW (Harpers Hill, Bringelly, Luddenham and Maitland Park) and Maui (subsp. *cuspidata*). In the invasive range of the African olive, individuals from Camden Park plus three individuals from Mount Annan (no 2, 8 and 17) and one from Bringelly (no 21) were assigned to both clusters C and E suggesting that they correspond to admixed individuals (i.e. hybrids between subspecies *europaea* and *cuspidata*). Two individuals from SA (Lonsdale no 16 and 22) also appear to be admixed with a percentage of assignment to cluster C of approximately 10–20%. The Mediterranean and African chloroplast lineages match with the two clusters as all invasive individuals assigned to cluster E and C with *P* > 0.8 show, respectively, a chlorotype of *europaea* and *cuspidata* (Fig. S3). In contrast, all individuals exhibiting Mediterranean chlorotypes in NSW (Camden Park, Bringelly no 21 and Mt Annan no 17) are admixed.

At *K* = 5, cultivars and invasive olives from South Australia are relatively well distinguished into two clusters (namely E_N_ and E_I_), while most individuals from South Africa, NSW and Maui are, respectively, assigned to a specific cluster (namely C_SA_, C_NSW_ and C_H_). Note that at *K* = 2, individuals from South Africa are not always assigned to cluster C with a high *P* value (Fig. 3). This is likely due to the strong genetic bottlenecks in invasive African olive populations (for instance, many alleles initially shared between *europaea* and *cuspidata* in the native range could have been lost after bottlenecks and may bias assignments). In contrast, at *K* = 5, no South African olive individuals are assigned to Mediterranean clusters with *P* > 0.05, while admixture between E and C clusters is still detected in the Camden Park population, one individual from Bringelly (no. 21), three individuals from Mt Annan (no. 2, 8 and 17) and two individuals from Lonsdale (no 16 and 22).

Pairwise *F*_ST_ between invasive populations confirmed the main patterns observed with the Bayesian clustering approach (Table S5). In particular, populations from South Australia and NSW were clearly differentiated (*F*_ST_ ranging from 0.28 to 0.33, when Camden Park is excluded). These high levels of differentiation likely reflect the initial differentiation between subspp. *cuspidata* and *europaea* (*F*_ST_ between Cape Town and Mediterranean cultivars is 0.23; *P* < 0.001). Yet, the genetic differentiation between Australian and Hawaiian populations of African olive exceeds 0.20 (Table S5) although all SSR alleles found in Maui were also detected in NSW (Table S4).

Low variation in plastid DNA was detected in the invasive populations of African olive (Fig. 1), and pairwise *F*_ST_ values were thus only estimated for South Australian populations. Based on this maternal marker, the genetic differentiation between South Australian populations ranged from 0.21 to 0.39 (Table S5). The high differentiation based on the plastid genome was unexpected as populations are in close proximity to each other (max. 16 km). On the three South Australian populations, the relative contribution of gene flow by pollen versus seeds (*r* = 49.8) indicates that dispersal of pollen is more efficient than seeds by several orders of magnitude at this small geographic scale.

### Genetic diversity of invasive and native populations based on nuclear SSRs

The eleven nuclear SSR loci used were polymorphic, but locus DCA15 was fixed in several populations of African olive from South Africa, NSW and Hawaii. For the ten loci that are polymorphic in both subspecies, the allelic richness revealed in Cape Town was significantly higher than in the set of 68 Mediterranean olive cultivars (Wilcoxon test: *P* < 0.05; Table 2). In contrast, in the invasive range, the South Australian population (subsp. *europaea*) displayed higher allelic richness than NSW populations (subsp. *cuspidata*) but the difference was not significant (Wilcoxon test: *P* = 0.16; Table 2). Compared with the putative native sources (Cape Town and Mediterranean cultivars), a reduction in allelic richness was found in both Australian invasive lineages (Wilcoxon tests: Cultivars/South Australia, *P* < 0.05; Cape Town/New South Wales, *P* < 0.01; Table 2); a reduction of 12.1% was revealed in the Mediterranean olive (South Australia), while it was of 57.5% in the African olive (NSW). The difference between Cape Town and NSW was also significant for *H*_S_ (*P* < 0.01; Table 2). Among NSW locations, the population from Camden Park displayed higher *R*_S_ and *H*_S_ values compared with all other populations (Table S7), but the difference between Camden Park and NSW (excluding admixed individuals) was significant only for *H*_S_ (Wilcoxon test: *P* < 0.05). In addition, the population from Maui (Hawaii) was particularly genetically impoverished (Table S7; see also Besnard et al. 2007a) and showed a significantly lower allelic richness and gene diversity that in African olives from NSW (Wilcoxon tests: *P* < 0.05 and *P* < 0.01, respectively).

**Table 2:** Allele size range (in bp), number of alleles (*N*_a_), allelic richness (*R*_S_ for 20 individuals), observed heterozygosity (*H*_O_), total diversity (*H*_S_) for each nuclear SSR locus for native and invasive trees of subspp. *europaea* (Mediterranean olive) and *cuspidata* (African olive).

	Mediterranean cultivars (native *europaea*)					South Australia* (invasive *europaea*)				
Locus	Allele size range	*N*_a_	*R*_S_	*H*_O_	*H*_S_	Allele size range	*N*_a_	*R*_S_	*H*_O_	*H*_S_
DCA1	208–272	12	6.48	0.75	0.64	208–278	8	5.67	0.78	0.74
DCA3	233–257	11	8.14	0.93	0.86	235–255	7	5.69	0.78	0.76
DCA5	195–215	9	6.95	0.46	0.47	195–209	8	6.72	0.77	0.76
DCA8	127–159	14	9.77	0.93	0.83	127–153	11	9.56	0.85	0.88
DCA9	163–209	16	11.13	0.88	0.85	163–213	14	10.06	0.85	0.87
DCA14	170–190	12	7.89	0.81	0.69	149–190	9	6.25	0.76	0.70
DCA15	247–271	7	4.76	0.77	0.65	247–271	5	4.44	0.71	0.72
DCA18	162–186	12	9.31	0.91	0.86	168–184	8	7.42	0.77	0.84
EMO3	213–226	10	7.92	0.93	0.81	213–226	8	6.55	0.79	0.82
GAPU71A	211–243	9	5.35	0.53	0.47	211–233	6	4.93	0.62	0.61
PA(ATT)2	106–124	6	5.29	0.82	0.77	106–124	6	5.87	0.77	0.78
Average†	–	11.1	7.82	0.80‡	0.73	–	8.5	6.87	0.77	0.78

	Cape Town (native *cuspidata*)					New South Wales* (invasive *cuspidata*)				
Locus	Allele size range	*N*_a_	*R*_S_	*H*_O_	*H*_S_	Allele size range	*N*_a_	*R*_S_	*H*_O_	*H*_S_
DCA1	214–278	20	20.00	0.90	0.95	214–260	8	6.52	0.77	0.77
DCA3	231–281	13	13.00	0.75	0.74	233–237	2	2.00	0.24	0.34
DCA5	196–202	3	3.00	0.40	0.48	200–202	2	2.00	0.33	0.32
DCA8	119–181	21	21.00	0.95	0.94	123–143	11	4.97	0.44	0.44
DCA9	167–215	12	12.00	0.80	0.83	167–237	14	10.35	0.80	0.87
DCA14	146–152	7	7.00	0.75	0.79	145–150	6	4.97	0.75	0.68
DCA15	247	1	1.00	–	–	247	1	1.00	–	–
DCA18	160–260	21	21.00	0.80	0.96	164–220	16	10.10	0.78	0.86
EMO3	200–219	14	14.00	0.90	0.93	207–213	5	4.48	0.60	0.67
GAPU71A	209–255	14	14.00	0.90	0.93	213–249	9	6.87	0.74	0.76
PA(ATT)2	100–118	5	5.00	0.40	0.39	100–118	4	3.00	0.46	0.45
Average†	–	13.0	13.00	0.76	0.79	–	7.7	5.53	0.59	0.62

* Excluding admixed individuals.

† DCA15 was not considered to compute average values because not variable in subsp. *cuspidata*.

‡ The *F*_IS_ value was significantly different from 0 only for cultivars (*F*_IS_ = −0.103; CI 95% = [−0.140 to −0.081]), for which the value was significantly negative. This result indicates heterozygous excess, probably due to human selection of early-generation admixed genotypes, maintained by clonal growth over long period of times.

### Demographic models and introduction scenarios

The ABC analyses, based on nuclear markers, allowed us to discriminate among the three tested introduction scenarios for the invasive African olive. From a total of 10^5^ simulations performed under each scenario, scenario 1 was selected with a posterior probability close to one hence favouring a sequential scenario with a first introduction into Australia followed by an introduction into Hawaii from Australia. The proportion of type-1 and type-2 errors were estimated (Table S8). Type-1 errors are around 0.14 for scenario 1, whereas the type-2 error ranged from 0.02 to 0.10. The results were similar for scenario 2, whereas both types of error were almost zero for scenario 3. These simulations show that the three scenarios are unlikely to be identified as the most probable scenario when they are indeed not the true scenarios and they are typically selected when they are the true scenarios. Altogether, this validation provides us with strong confidence in our model choice results.

**Figure 4:**
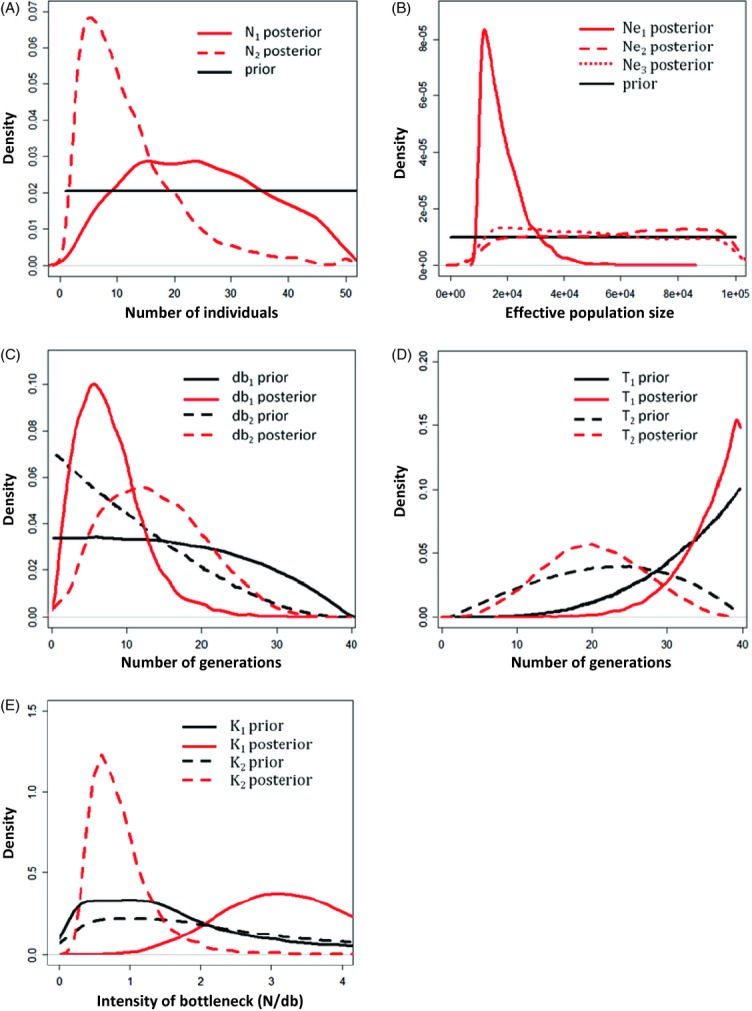
Prior and posterior density curves for all demographic parameters. All posterior and prior densities were computed with DiyABC version 2.0 (Cornuet et al. submitted) and were estimated from 500 000 and 5000 samples, respectively (i.e. the best 1% of the 500 000 simulated data). A. Effective numbers of founding individuals in the invasive range (N_1_ and N_2_); B. Effective population size in number of individuals in both native (Ne_1_) and invasive (Ne_2_ and Ne_3_) ranges; (C) Latency phase after introduction in the invasive range (db_1_ and db_2_; in number of generations); (D) Number of generations since introduction to NSW, Australia (T_1_), and since second introduction to Maui, Hawaii (T_2_); E. Severity of the bottleneck during the first and the second events of introduction (K_1_ and K_2_).

Because scenario 1 was identified as the most probable, posterior distributions for the parameters of interest were inferred for this scenario only. Figure 4 shows the priors and posteriors for all parameters, and Table 1 provides the mean, median and mode estimated for these distributions. For some parameters, the posterior differs noticeably from the prior (e.g. N_2_, N_e1_; Fig. 4A,B). This suggests that the genetic data contain substantial information to estimate these demographic parameters. For other parameters (e.g. N_e2_; Fig. 4B), little information seems to be provided beyond that present in the prior. We focus on modal values below, but are aware that the distributions are sometimes wide and that no single estimate (mean, median or mode) fully summarizes our results.

As Fig. 4A shows, the posterior for N_1_ is not very different from the prior, but tends to exclude extreme values and favour the central values with a mode around 20–25 individuals introduced to Australia. For N_2_, the situation is different with small values clearly having a stronger support, suggesting that less than ten individuals were introduced into Hawaii. For the bottleneck severity in Australia (K_1_), the posterior distribution favours values close to 3 and displays a peak at 3.10. For Hawaii (K_2_), the posterior distribution displays values lower than 1 and shows a peak at 0.59 indicating a more severe bottleneck than in Australia (Fig. 4E). For the current population effective size parameters (N_e1_, N_e2_ and N_e3_; Fig. 4B), the posterior distributions show different patterns. For the South African population (N_e1_), the analysis suggests that values on the lower end of the distributions are more likely, whereas for the Australian and Hawaiian populations (N_e2_ and N_e3_), the posteriors are very wide and provide no clear information. Regarding the ‘latency phase’ or duration of the bottleneck, the results are difficult to interpret. In Australia, the db_1_ posterior exhibits a clear peak at the lower end (a modal value between 2 and 6) and stronger support compared with the prior for most values below 10–15 generations, hence supporting a rather short ‘latency phase’ if any (Fig. 4C). In Hawaii, however, the posterior is shifted towards larger values compared with the prior, with a modal value around 12 generations.

For T_1_, the time at which olives were introduced in Australia, we find a mode around 39 generations, which is close to the upper limit of our prior (Fig. 4D). The two distributions (prior and posterior) are similar, but the posterior still seems to provide no support for values below 20 generations (with a mean generation time of 5 years, that would correspond to 100 years) and thus to favour an old rather than a recent event. The second introduction time (T_2_, to Hawaii) exhibits a posterior which is also not very different from the prior and seems to favour slightly more recent event compared with the prior, perhaps around 19 generations ago but caution is clearly required here (Fig. 4D).

Note that we obtained similar results with the second ABC approach. This is described and discussed in the supplementary information (Data S1).

### Precision on parameter estimations, sensitivity to priors and robustness of inference

Several measures of bias and error were computed from pseudo-observed data. We found a positive bias for most parameters with small values (< 0.1) for the split times (T_1_ and T_2_), values between 0.1 and 0.3 for the number of founders (N_1_ and N_2_) and N_e1_, and higher values (between 0.3 and 0.5) for N_e2_ and N_e3_ (Table S9). The values of the root of the relative mean square error (RRMSE) followed the same trend with larger errors for N_e2_ and N_e3_ and smaller RRMSE values for T_1_ and T_2_ which were therefore reasonably estimated (Table S10). Table S11 and Fig. S4 show the results of the model checking computation. The PCA representation of model checking exhibits a rather good recovery of the posterior predictive distribution and the observed data, showing a certain confidence in the ‘goodness of fit’ of our inference (Fig. S4). Numerical results indicate, however, a substantial excess of test statistics showing probabilities in the tail areas (i.e. 6 over 24 test statistics; Table S11). This suggests that the selected scenario and associated posterior distributions are not that well corroborated by the observed data and hence that our model-posterior combination probably misses some aspects of the real evolutionary history. When we used different priors, some of the results changed, whereas some parameters provided similar posteriors (Table S12). For instance, we found limited effect on the posterior distributions for the number of founders (N_1_ and N_2_), the severity of bottleneck (K_1_ and K_2_) and the split times (T_1_ and T_2_), whereas some effects could be noted on the three current effective size parameters (N_e1_, N_e2_ and N_e3_). Altogether, this suggests that while some of our results should be interpreted with care (especially the effective size of populations), the results on N_1_, N_2_, K_1_, K_2_, T_1_ and T_2_ may be more reliable.

## Discussion

### Two olive taxa have been introduced in the invasive range

Our genetic analysis confirmed that all invasive olive populations sampled until now originated from two distinct taxa, as first suggested by Besnard et al. (2007a) on a smaller tree sample. First, the cpDNA variation (Fig. 1) shows that South Australian olive populations have three Mediterranean chlorotypes (which are detected in Mediterranean olive cultivars; Besnard et al. 2011), while populations from NSW (with the exception of Camden Park, Mount Annan no. 17 and Bringelly no. 21) and Maui display two African chlorotypes. Interestingly, African olive individuals from northern New Zealand (Kermadec, Auckland Bay), Norfolk and Saint Helena exhibit the same two chlorotypes as NSW and Maui populations, suggesting a common origin. These two chlorotypes are closely related (Fig. 2 and Fig. S2), and in the native range, they were only detected in the Cape Town population. The introduction of African olive from South Africa to the invasive range is strongly supported by these data. Second, the Structure analysis based on nuclear SSRs (Fig. 3) confirms that invasive olive populations in South Australia and NSW are closely related to Mediterranean cultivars and South African Olives, respectively. These results indicate a strong congruence between plastid and nuclear genetic patterns although gene dispersal by pollen is more efficient than by seeds by several orders of magnitude (i.e. about 50 times in South Australian populations). Genetic data are thus powerful tools to trace the invasion of the two olive lineages, particularly to test for admixture events (see below).

### Admixture between the two olive subspecies can be locally high

In South Australia, we initially suspected that Shepherds Hill could be a putative site of simultaneous introductions for both Mediterranean and African olives. Indeed, while the Mediterranean subspecies is highly invasive in South Australia, the African olive has been reported to have also naturalized at Shepherds Hill (in Cuneo and Leishman 2006). Although no early admixture event has been detected at this location, two hybrids of early generation were detected at Lonsdale (located at approximately 12 km from Shepherds Hill) confirming the initial hypothesis that the African subspecies has naturalized in this region and exchanged genes with the Mediterranean olive in South Australia.

In NSW, our results indicate that Camden Park is a hybrid population as all individuals display alleles from subspp. *cuspidata* and *europaea*. The occurrence of two chlorotypes suggests that at least two cultivars of subspecies *europaea* have been involved in the constitution of the Camden Park population. The population of Camden Park should be relatively old if we consider the size of trees, and hybridization may have occurred during the first steps of the African olive invasion in NSW. Camden Park is highly differentiated from other East Australian populations, despite close proximity to some of them (Table S5). This could be due to some limitations for gene flow between Camden Park and other populations. Particularly, chlorotype E3.2 is very frequent in Camden Park (23/25), and our observations confirmed that this cytoplasm is associated with male sterility (see Besnard et al. 2000). This means that pollen gene flow from Camden Park is highly reduced. The lack of dispersal for pollen coupled with low capacity of fruit dispersal (as shown here in South Australian populations) may contribute to the highly reduced gene flow from Camden Park to other invasive populations. Only three early-generation hybrids were detected at Mount Annan, a NSW population only 4–5 km away from Camden Park, and clearly, panmixia is not attained between these two adjacent populations.

Species distribution modelling has recently suggested that the current habitat in NSW is more suitable for the African olive, while habitat in South Australia is more suitable for the Mediterranean olive (J. Cornuault, A. Khimoun, P. Cuneo and G. Besnard, in preparation). We can thus suspect that subsp. *cuspidata* is not well adapted to South Australia and subsp. *europaea* is not well adapted to NSW, consistent with our finding that these two subspecies are dominant in the location predicted to be optimal. This means that ecological requirements may drive the local success of each olive subspecies. Due to the dominance of one taxon over the other, early-generation hybrids are expected to be rare on the front of invasion, and advanced generations, if any, are expected to be backcrossed to the dominant subspecies.

### Evidence for genetic erosion due to recurrent bottlenecks in the invasive African olive range

Plastid DNA data indicate that the invasive African olive has been introduced from South Africa (see above). In addition, all nuclear SSR alleles detected in Maui were also present in NSW, suggesting that NSW could have been a source for secondary invasions. Our ABC analysis gave a strong posterior support to this scenario with sequential introductions (Fig. S1, Table S8) and rejected independent colonization of Australia and Hawaii. Thus, the previous statement of Besnard et al. (2007a) of a sequential colonization of Hawaii from Australia is validated.

Compared with putative native sources, both invasive olive subspecies have experienced a significant reduction in diversity in Australia (Table 2), but a stronger bottleneck was detected in NSW than South Australia (see also Besnard et al. 2007a). This may be due to an introduction of a limited number of trees from South Africa (from at least two mother trees as indicated by the presence of two distinct African chlorotypes). Consistently, both ABC analyses indicate that the number of founder trees in NSW was less than 30 individuals, and the secondary invasion to Hawaii resulted from an even more reduced number of founder trees from NSW (less than 10 individuals). Our simulations also indicate that the bottleneck was more severe in Hawaii (K = 0.59) than in Australia (K = 3.10; Table 1). The estimates of the number of generations since introduction supported an early introduction in NSW (T_1 _> 35 generations) and a more recent introduction in Hawaii (T_2_ < 20 generations) and this was supported by the two ABC analyses that we performed (See Data S1). Altogether, congruence between the two different ABC approaches used, and the robustness of posterior to changes in the priors suggests a certain confidence in our results and shows the utility of comparing several methods to validate inferences. Our results also suggest that the mean generation time is relatively short in the invaded range (approximately 5 years, considering an initial introduction to NSW about 200 years ago).

While we strongly support the use of model-based approaches such ABC modelling, we would like to point at several limitations of the ABC approach as applied here and elsewhere. The first is that DiyABC and in fact most population genetics models assume nonoverlapping generations, which is unlikely to hold for long-living organism such as the olive tree. How this would affect our results is unclear, but it suggests that a specific modelling framework should be developed to study the effect of long-living organisms on the estimation of population genetics parameters. Another related issue is that both DiyABC and *ms* approaches ignore the fact that the introduction of one olive tree may actually correspond to the introduction of one female fertilized by several males (due to the introduction either of trees bearing fruits, or seed sets collected on a few mothers). This means that the number of founders estimated here and the values obtained for several parameters should be considered cautiously. Altogether, we believe that our results should be taken as a first step towards a better understanding of the details of the invasion of several regions by the olive.

### Concluding remarks and recommendations on the invasive olive management

An important result of our study is to conclusively resolve the issue of *O. europaea* hybridization raised by Breton et al. (2008), who stated that natural hybridizations between *europaea* and *cuspidata* subspecies were very unlikely to occur in Australia. Here, we have strong evidence for hybridization in early introduction sites in both NSW and South Australia. Hybridization is putatively an important process during the olive invasion. This phenomenon could have reduced the negative effects linked to the loss of genetic diversity that occurred via successive bottlenecks during the initial colonization events and also helped populations to better adapt to new environments (Ellstrand and Schierenbeck 2000; Figueroa et al. 2003; Facon et al. 2006; Keller and Taylor 2010). Additional investigations are necessary to determine whether trees at the invasion front represent introgressed genomes and are responsible for adaptation to local conditions. In addition, with the increased cultivation of subspecies *europaea* throughout eastern Australia, there is increased potential for hybridization and new recombinations with existing invasive populations of subspecies *cuspidata*. The impact of this phenomenon on the olive invasiveness could be also assessed in the future.

Considering another practical aspect, it is also important to note that low population size and successive bottlenecks did not preclude olive invasion, particularly on the Hawaiian archipelago. Such phenomenon has been already reported on other invasive organisms and could allow a rapid evolution of adaptive traits (e.g. Dlugosch and Parker 2008). For the management of invasive populations, our study shows the necessity to take rapid decision to stop or limit invasion. Even with a very small introduced population, of the order of ten individuals (and probably fewer), the risk of an invasion in large scale is real and deserves to be considered.

## Data accessibility

Sample locations and microsatellite genetic profiles are available in Supplementary Information (Tables S1 to S4).

## Supporting Information

**Data S1**. Alternative ABC implementation using in-house scripts, the ms program and the abc R package. **Figure S1**. The three demographic scenarios of African olive invasion in Australia and Hawaii. **Figure S2**. Reduced-median networks of haplotypes detected in the native range of subsp. cuspidata. **Figure S3**. Barplot of the STRUCTURE analysis for the Australian and Hawaiian invasive olive populations based on the most probable number of genetic clusters K = 2. **Figure S4**. PCA of the model checking computation in the space of summary statistics. **Table S1**. Geographic origins of native and invasive samples characterized with cpDNA markers. **Table S2**. Geographic origins of the 68 Mediterranean cultivars characterized with nuclear SSR markers. **Table S3**. Profile and geographic origin of 39 chlorotypes detected in the native range of subsp. *cuspidata* compared to the three chlorotypes shared between Mediterranean cultivars and invasive olives. **Table S4**. Nuclear microsatellite dataset for invasive populations. **Table S5**. Pairwise genetic differentiation (F_ST_, in percent) between invasive populations based on nuclear SSRs. **Table S6**. Number of alleles (N_a_), allelic richness (R_S_), observed and expected heterozygosities (H_O_ and H_S_ respectively) for each SSR locus for the three invasive populations of subsp. *europaea*. **Table S7**. Number of alleles (N_a_), allelic richness (R_S_), observed and expected heterozygosities (HO and HS respectively) for each SSR locus for the six invasive populations of subsp. *cuspidata*. **Table S8**. Type-1 and type-2 error rates for simulated data. **Table S9**. Precision on parameter estimations based on 500 pseudoobserved data sets (pods) with the measure of the relative average bias, estimated under scenario 1. **Table S10**. Precision on parameter estimations based on 500 pseudoobserved data sets (pods) with the measure of the square Root of the Relative Mean Square Error (RRMSE) and the Relative Median Absolute Deviation (RMedAD), estimated under scenario 1. **Table S11**. Model checking for introduction scenario 1. **Table S12**. Effect of different priors on the posteriors for the real data sets.
