# Supplementary material for: History of the invasive African olive tree in Australia and Hawaii: evidence for sequential bottlenecks and hybridization with the Mediterranean olive
Source: Evol Appl. 2013 Nov 28;7(2):195–211. doi: 10.1111/eva.12110 (PMC3927883; doi:10.1111/eva.12110)
Supplement: Supplementary file 1 — Data S1. Alternative ABC implementation using in-house scripts, the ms program and the abc R package. Figure S1. The three demographic scenarios of African olive invasion in Australia and Hawaii. Figure S2. Reduced-median networks of haplotypes detected in the native range of subsp. cuspidata. Figure S3. Barplot of the STRUCTURE analysis for the Australian and Hawaiian invasive olive populations based on the most probable number of genetic clusters K = 2. Figure S4. PCA of the model checking computation in the space of summary statistics. Table S1. Geographic origins of native and invasive samples characterized with cpDNA markers. Table S2. Geographic origins of the 68 Mediterranean cultivars characterized with nuclear SSR markers. Table S3. Profile and geographic origin of 39 chlorotypes detected in the native range of subsp. cuspidata compared to the three chlorotypes shared between Mediterranean cultivars and invasive olives. Table S4. Nuclear microsatellite dataset for invasive populations. Table S5. Pairwise genetic differentiation (FST, in percent) between invasive populations based on nuclear SSRs. Table S6. Number of alleles (Na), allelic richness (RS), observed and expected heterozygosities (HO and HS respectively) for each SSR locus for the three invasive populations of subsp. europaea. Table S7. Number of alleles (Na), allelic richness (RS), observed and expected heterozygosities (HO and HS respectively) for each SSR locus for the six invasive populations of subsp. cuspidata. Table S8. Type-1 and type-2 error rates for simulated data. Table S9. Precision on parameter estimations based on 500 pseudoobserved data sets (pods) with the measure of the relative average bias, estimated under scenario 1. Table S10. Precision on parameter estimations based on 500 pseudoobserved data sets (pods) with the measure of the square Root of the Relative Mean Square Error (RRMSE) and the Relative Median Absolute Deviation (RMedAD), estimated under scenario 1. Table S11. Mo [file eva0007-0195-sd1.docx]

**Supplementary information**

**History of the invasive African olive tree: evidence for sequential bottlenecks and hybridizations with the Mediterranean olive**

**G. Besnard & J. Dupuy et al.**

**Supplementary information includes:**

**Table S1.** Geographic origins of native and invasive samples characterized with cpDNA markers

**Table S2.** Geographic origins of the 68 Mediterranean cultivars characterized with nuclear SSR markers

**Table S3.** Profile and geographic origin of 39 chlorotypes detected in the native range of subsp. *cuspidata* compared to the three chlorotypes shared between Mediterranean cultivars and invasive olives

**Table S4.** Nuclear microsatellite dataset for invasive populations

**Table S5.** Pairwise genetic differentiation (*F_ST_*, in percent) between invasive populations based on nuclear SSRs

**Table S6.** Number of alleles (*N_a_*), allelic richness (*R_S_*), observed and expected heterozygosities (*H_O_* and *H_S_* respectively) for each SSR locus for the three invasive populations of subsp. *europaea*

**Table S7.** Number of alleles (*N_a_*), allelic richness (*R_S_*), observed and expected heterozygosities (*H_O_* and *H_S_* respectively) for each SSR locus for the six invasive populations of subsp. *cuspidata*

**Table S8.** Type-1 and type-2 error rates for simulated data

**Table S9.** Precision on parameter estimations based on 500 pseudo-observed data sets (pods) with the measure of the relative average bias, estimated under scenario 1

**Table S10.** Precision on parameter estimations based on 500 pseudo-observed data sets (pods) with the measure of the square Root of the Relative Mean Square Error (RRMSE) and the Relative Median Absolute Deviation (RMedAD), estimated under scenario 1

**Table S11.** Model checking for introduction scenario 1

**Table S12.** Effect of different priors on the posteriors for the real data sets

**Figure S1.** The three demographic scenarios of African olive invasion in Australia and Hawaii

**Figure S2.** Reduced-median networks of haplotypes detected in the native range of subsp. *cuspidata*

**Figure S3.** Barplot of the Structure analysis for the Australian and Hawaiian invasive olive populations based on the most probable number of genetic clusters *K* = 2

**Figure S4.** PCA of the model checking computation in the space of summary statistics

**Supplemental Methods.** Alternative ABC implementation using in-house scripts, the ms program and the abc R package

**References**

**Table S1.** Geographic origins of native and invasive samples characterized with cpDNA markers. Populations also characterized with nuclear markers are in bold. N represents the number of individuals sampled for each location. The cpDNA lineages (cpDNA) found in subsp. *cuspidata* are given for each provenance (according to Besnard et al. 2007b). Herbarium specimens are indicated in brackets.

| Location | Country | N | Lat | Long | cpDNA |
| --- | --- | --- | --- | --- | --- |
| - Native range (subsp. *cuspidata*): |  |  |  |  |  |
| Cape Town* | **South Africa** | **20** | **-33.986** | **18.423** | **A** |
| Amalundu, Bulawayo | Zimbabwe | 1 | -20.250 | 28.700 | A |
| Victoria Falls [C.F.H. Monro 367 (BM)] | Zimbabwe | 1 | -17.961 | 25.855 | A |
| Inhaca Island [A.O.D. Mogg 30898 (K)] | Mozambique | 1 | -26.038 | 32.963 | A |
| Dos d'Ane, Cirque de Mafate | Reunion | 7 | -20.986 | 55.373 | A |
| Baie du Cap | Mauritius | 1 | -20.48 | 57.371 | A |
| Rumphi, Nyika [J.D. Chapman 6305 (K)] | Malawi | 1 | -10.65 | 33.888 | A |
| Arusha, Mt Longido [G. Simon *et al.* 432 (K)] | Tanzania | 1 | -2.696 | 36.698 | A |
| Nairobi | Kenya | 2 | -1.411 | 36.645 | A |
| Timau, Mt Kenya | Kenya | 11 | -0.011 | 37.265 | A |
| Mt Elgon | Kenya | 5 | 1.185 | 34.665 | A |
| Mt Kulal [B. Verdcourt 2247 (K)] | Kenya | 1 | 2.750 | 36.935 | A |
| Dandu, Northern Province [J.B. Gillett 13422 (K)] | Kenya | 1 | 3.433 | 39.900 | C2 |
| Hamassen Province: Coasien [A. Pappi 3618 (BM)] | Erythrea | 1 | 14.733 | 39.817 | C2 |
| Assaorta Province: Zazegá [A. Pappi 5028 (BM)] | Erythrea | 1 | 15.017 | 39.400 | C2 |
| Mt Bizen [P.R.O. Bally 19 (K)] | Erythrea | 1 | 15.331 | 39.084 | C2 |
| Motulu, ESE Amba Alagi [H.F. Mooney 6718 (K)] | Ethiopia | 1 | 12.968 | 39.567 | C2 |
| Shoa (šewā) Province, West of Addis Abeba [F.G. Meyer 8177 (K)] | Ethiopia | 1 | 9.219 | 38.583 | C2 |
| Ahmar Mtns, 95 km W of Dire Dawa [W. Burger 1643 (K)] | Ethiopia | 1 | 9.250 | 41.217 | C2 |
| Gara Mulatta Mts, 20 km of Alemaya [W. de Wilde 9902 (K)] | Ethiopia | 1 | 9.267 | 41.733 | C2 |
| Lower altitudes of Wagger Mts [P.E. Glover & H.B. Gilliland 475 (BM)] | Somalia | 1 | 9.878 | 44.828 | C2 |
| Sheekh, Gaan Libah, Togdheer [O.J. Hansen *et al.* 6426 (K)] | Somalia | 1 | 10.009 | 45.458 | C2 |
| Misan road, 45 km SW of Taif [S.S. Collenette 8524 (K)] | Saudi Arabia | 1 | 21.083 | 40.263 | C2 |
| Al Mahwit | Yemen | 6 | 15.481 | 43.581 | C2 |
| Jebel Qumar, Drofar [R.M. Lawton 2256 (K)] | Oman | 1 | 16.677 | 53.101 | C1 |
| Jebel Akhdar [A. Radcliffe-Smith 4035 (K)] | Oman | 1 | 23.369 | 57.916 | C1 |
| Asmar, Barikot [L. Edelberg 1630 (K)] | Afghanistan | 1 | 35.030 | 71.354 | C1 |
| Bashgal-Tal (Darrah-i-Katigal) Nuristan, 12 km Barikot, Prov. Kunar [D. Poldech 16769 (K)] | Afghanistan | 1 | 35.190 | 71.490 | C1 |
| Kerman province | Iran | 6 | 28.156 | 57.063 | C1 |
| Guangzhou (ornamental trees) | China | 2 | 23.082 | 113.261 | C1 |

Herbarium origin: (K) = Kew; (BM) = British Museum of Natural History; * populations previously characterized with eight nuclear microsatellites (Besnard et al. 2007a).

**Table S1 (end).**

| Location | Country | N | Lat | Long | cpDNA |
| --- | --- | --- | --- | --- | --- |
| - Native range (subsp. *europaea*): |  |  |  |  |  |
| Wild and cultivated Mediterranean trees (see Besnard et al. 2013)^#^ | Mediterranean area | 1197 | - | - | E1/E2/E3 |
|  |  |  |  |  |  |
| - Invasive range (subsp. *cuspidata*) : |  |  |  |  |  |
| Mount Annan, Campbelltown, NSW (Cumberland Plain) | **Australia** | **25** | **-34.078** | **150.761** | **A/E3** |
| Bringelly, Campbelltown, NSW (Cumberland Plain)* | **Australia** | **29** | **-33.955** | **150.712** | **A/E1** |
| Luddenham, Campbelltown, NSW (Cumberland Plain) | **Australia** | **25** | **-33.889** | **150.699** | **A** |
| Maitland Park, Maitland, NSW (Central Hunter region) | **Australia** | **5** | **-32.742** | **151.556** | **A** |
| Harpers Hill, Maitland, NSW (Central Hunter region) | **Australia** | **24** | **-32.705** | **151.417** | **A** |
| Ravensworth, Maitland, NSW(Central Hunter region) | **Australia** | **6** | **-32.440** | **151.056** | **A** |
| Norfolk Island [G. Uhe 1190 (K)] | Australia | 1 | -29.040 | 167.950 | A |
| Raoul Island [W.R. Sykes 214 (K)] | New Zealand | 1 | -29.270 | -177.930 | A |
| Auckland Bay [R. Melville 5373 (K)] | New Zealand | 1 | -36.330 | 174.700 | A |
| Maui* | **Hawaii, USA** | **26** | **20.840** | **-156.310** | **A** |
| Longwood [P.W. Lambdon & A. Darlow SH032 (K)] | Saint Helena | 1 | -15.950 | -5.687 | A |
|  |  |  |  |  |  |
| - Invasive range (subsp. *europaea*): |  |  |  |  |  |
| Brownhill Creek, SA (Adelaide Hills)* | **Australia** | **29** | **-34.985** | **138.626** | **E1/E3** |
| Shepherds Hill, SA (Adelaide Hills) | **Australia** | **20** | **-35.013** | **138.585** | **E1/E2/E3** |
| Lonsdale, SA (Adelaide Hills)* | **Australia** | **30** | **-35.093** | **138.497** | **E1/E2/E3** |
|  |  |  |  |  |  |
| - Invasive range (undetermined subspecies): |  |  |  |  |  |
| Camden Park, Campbelltown, NSW (Cumberland Plain) | **Australia** | **25** | **-34.059** | **150.694** | **E3/E1** |

Herbarium origin: (K) = Kew; (BM) = British Museum of Natural History; * populations previously characterized with eight nuclear microsatellites (Besnard et al. 2007a); ^#^ 68 cultivated accessions were also characterized with nuclear SSR markers (see Table S2).

**Table S2.** Geographic origins of the 68 Mediterranean cultivars characterized with nuclear SSR markers. The plastid haplotype (cpDNA) is given for each accession.

| Cultivar names | Country | Material origin | cpDNA |
| --- | --- | --- | --- |
| Kaissy | Syria | OWGB | E1-1 |
| Zaity | Syria | OWGB | E1-2 |
| Abbadi | Syria | OWGB | E1-1 |
| Chalchali | Syria | OWGB | E1-1 |
| Chami | Syria | OWGB | E1-1 |
| Souri | Lebanon | OWGB | E1-1 |
| Merhavia | Israel | OWGB | E1-1 |
| Nabali | Israel | OWGB | E1-1 |
| Hamed | Egypt | OWGB | E1-1 |
| Wardan | Egypt | OWGB | E1-1 |
| Ladolia | Cyprus | Field Collect | E1-1 |
| Belluti | Turkey | OWGB | E1-1 |
| Vallanolia | Turkey | OWGB | E1-1 |
| Trylia | Turkey | OWGB | E1-1 |
| Uslu | Turkey | OWGB | E1-1 |
| Ayvalik | Turkey | OWGB | E1-1 |
| Memecik | Turkey | OWGB | E1-1 |
| Mastoidis | Greece | OWGB | E1-1 |
| Mirtolia | Greece | OWGB | E1-1 |
| Kalamon | Greece | OWGB | E1-1 |
| Konservolia | Greece | OWGB | E1-1 |
| Megaritiki | Greece | OWGB | E2-2 |
| Lastovka | Greece | OWGB | E1-1 |
| Moraiolo | Italy | OWGB | E1-1 |
| Nocellara Belica | Italy | OWGB | E1-1 |
| Frantoio | Italy | OWGB | E1-1 |
| Giaraffa | Italy | OWGB | E1-2 |
| Pizz'e carroga | Italy | OWGB | E1-1 |
| Carolea | Italy | OWGB | E1-2 |
| Nocellara | Italy | OWGB | E1-1 |
| Itrana | Italy | OWGB | E1-1 |
| Bosana | Italy | OWGB | E1-1 |
| Confetto | Italy | OWGB | E1-1 |
| Picholine | France | OWGB | E2-1 |
| Cailletier | France | INRAM | E1-1 |
| Olivière | France | INRAM | E3-1 |
| Zinzala | France, Corsica | Field Collect | E2-2 |
| Capanacce | France, Corsica | Field Collect | E1-1 |
| Sabina | France, Corsica | Field Collect | E2-4 |
| Galega | Portugal | OWGB | E1-2 |
| Empeltre | Spain | OWGB | E1-1 |
| Lechin de Granada | Spain | OWGB | E1-1 |
| Picudo | Spain | OWGB | E1-1 |
| Changlot Real | Spain | OWGB | E1-1 |
| Blanqueta | Spain | OWGB | E1-3 |
| Villalonga | Spain | OWGB | E1-3 |
| Arbequina | Spain | OWGB | E1-1 |
| Gordal Sevillana | Spain | OWGB | E1-2 |
| Picual | Spain | OWGB | E1-1 |
| Cornicabra | Spain | OWGB | E1-1 |
| Hojibianca | Spain | OWGB | E1-1 |
| Lechin de Sevilla | Spain | OWGB | E2-3 |
| Verdial de Huevar | Spain | OWGB | E1-1 |
| Verdial de Velez | Spain | OWGB | E1-1 |
| Manzanilla de Almeria | Spain | OWGB | E1-1 |

**Table S2 (end).**

| Cultivar names | Country | Material origin | cpDNA |
| --- | --- | --- | --- |
| Negrillo de la Carlota | Spain | OWGB | E1-1 |
| Pico Limon | Spain | OWGB | E1-1 |
| Manzanilla Cacerana | Spain | OWGB | E1-1 |
| Manzanilla de Sevilla | Spain | OWGB | E1-1 |
| Taksrit | Algeria | Field Collect | E1-1 |
| Chemlal | Algeria | OWGB | E3-2 |
| Zarrazzi | Tunisia | OWGB | E3-2 |
| Zalmati | Tunisia | OWGB | E1-1 |
| Meski | Tunisia | OWGB | E1-1 |
| Chemchali-Jemri | Tunisia | OWGB | E1-1 |
| Chétoui | Tunisia | OWGB | E1-2 |
| Chemlali | Tunisia | OWGB | E1-2 |
| Picholine Marocaine | Morocco | OWGB | E1-1 |

**Table S3.** Profile and geographic origin of 39 chlorotypes detected in the native range of subsp. *cuspidata* compared to the three chlorotypes shared between Mediterranean cultivars and invasive olives. The number of occurrences in the native accessions is given. Alleles of each locus are coded for the reduced-median network analysis (Figure S2). Stretch size of the repeated motif is given for each microsatellite locus, while a binary code (0/1) has been defined for indels and single nucleotide. This coding was verified by sequencing alleles of a few chlorotypes (Besnard et al. 2011) and two alleles with an unusual size at locus 7. The complete dataset for all wild olive samples is available upon request to the corresponding author.

* Haplotype profiles for which a complete chloroplast sequence is available (Besnard et al. 2011); ^a^ EMBL accessions no HF564609 & HF564610; ^b^ For Mediterranean haplotypes, the number of occurrences in the native range is based on the characterization of 534 cultivars (Besnard et al. 2013)

# Table S4. Nuclear microsatellite dataset for invasive populations (11 SSR loci). Each allele is identified by its size in bp.

| **Sample code** | **DCA05** | | **DCA18** | **Gapu 71A** | **DCA14** | **PA(ATT)2** | **DCA08** | **DCA09** | **DCA01** | **DCA15** | **DCA03** | **EMO3** |
| --- | --- | --- | --- | --- | --- | --- | --- | --- | --- | --- | --- | --- |
| **Southern Australia:** | |  |  |  |  |  |  |  |  |  |  |  |
| Shepherd Hill 1 | **205-207** | | **172-180** | **215-215** | **182-184** | **106-109** | **131-131** | **177-195** | **208-218** | **258-268** | **235-235** | **220-223** |
| Shepherd Hill 2 | **201-205** | | **180-184** | **211-211** | **184-190** | **109-115** | **141-145** | **173-195** | **229-276** | **258-268** | **247-251** | **220-223** |
| Shepherd Hill 3 | **205-207** | | **176-180** | **211-215** | **190-190** | **115-115** | **133-133** | **163-185** | **208-270** | **247-268** | **245-251** | **223-225** |
| Shepherd Hill 4 | **207-207** | | **178-180** | **215-215** | **173-190** | **109-112** | **127-137** | **163-183** | **208-276** | **258-268** | **245-245** | **223-223** |
| Shepherd Hill 5 | **199-201** | | **170-172** | **213-215** | **173-190** | **106-112** | **133-145** | **195-195** | **208-218** | **258-258** | **241-245** | **220-225** |
| Shepherd Hill 6 | **199-203** | | **170-176** | **211-215** | **173-190** | **108-112** | **127-127** | **163-177** | **208-276** | **247-247** | **235-241** | **218-221** |
| Shepherd Hill 7 | **207-207** | | **172-176** | **213-215** | **184-190** | **112-115** | **133-137** | **177-207** | **208-208** | **258-268** | **251-251** | **218-226** |
| Shepherd Hill 8 | **199-207** | | **168-182** | **215-233** | **173-184** | **106-115** | **127-149** | **205-207** | **208-244** | **258-268** | **241-251** | **213-223** |
| Shepherd Hill 9 | **201-205** | | **180-184** | **211-215** | **184-190** | **109-115** | **131-141** | **183-195** | **229-276** | **247-268** | **235-247** | **220-221** |
| Shepherd Hill 10 | **199-203** | | **168-170** | **233-233** | **173-190** | **108-115** | **127-137** | **163-177** | **208-208** | **258-258** | **235-241** | **218-223** |
| Shepherd Hill 11 | **195-205** | | **180-182** | **211-211** | **184-184** | **106-115** | **133-145** | **163-211** | **218-276** | **258-268** | **235-247** | **218-218** |
| Shepherd Hill 12 | **199-203** | | **168-170** | **211-233** | **173-173** | **108-112** | **137-149** | **163-205** | **208-276** | **247-268** | **235-241** | **218-221** |
| Shepherd Hill 13 | **199-203** | | **176-182** | **215-233** | **173-190** | **108-112** | **127-127** | **205-211** | **244-276** | **258-268** | **235-241** | **218-221** |
| Shepherd Hill 14 | **201-207** | | **172-172** | **213-215** | **157-178** | **115-115** | **139-139** | **163-177** | **208-218** | **247-268** | **241-241** | **220-222** |
| Shepherd Hill 15 | **199-205** | | **172-180** | **211-215** | **173-190** | **108-115** | **127-139** | **167-207** | **208-218** | **247-258** | **241-251** | **213-220** |
| Shepherd Hill 16 | **199-207** | | **168-170** | **211-215** | **173-190** | **112-115** | **127-139** | **167-207** | **244-270** | **268-268** | **235-247** | **220-221** |
| Shepherd Hill 17 | **199-207** | | **172-176** | **211-215** | **180-190** | **115-115** | **139-139** | **167-207** | **208-208** | **247-268** | **241-251** | **218-225** |
| Shepherd Hill 18 | **199-199** | | **168-180** | **215-235** | **173-190** | **106-115** | **127-131** | **177-207** | **218-276** | **258-271** | **235-235** | **221-225** |
| Shepherd Hill 19 | **203-207** | | **182-182** | **213-233** | **173-178** | **115-124** | **139-149** | **163-163** | **208-218** | **268-268** | **241-241** | **220-225** |
| Shepherd Hill 20 | **201-205** | | **178-180** | **215-233** | **172-173** | **112-115** | **127-139** | **167-177** | **208-276** | **247-258** | **235-251** | **218-221** |
| Brownhill Creek 1 | **199-201** | | **170-176** | **211-215** | **173-178** | **109-115** | **133-139** | **167-177** | **208-276** | **268-271** | **241-251** | **220-221** |
| Brownhill Creek 2 | **199-207** | | **170-182** | **215-215** | **184-190** | **109-115** | **127-131** | **195-207** | **218-276** | **258-258** | **247-251** | **218-218** |
| Brownhill Creek 3 | **199-207** | | **178-182** | **213-215** | **190-190** | **115-115** | **131-139** | **183-207** | **208-218** | **247-258** | **245-247** | **218-223** |
| Brownhill Creek 4 | **199-201** | | **168-182** | **215-215** | **173-190** | **109-109** | **131-139** | **167-207** | **216-218** | **247-247** | **241-241** | **221-223** |
| Brownhill Creek 5 | **205-207** | | **180-180** | **215-215** | **190-190** | **115-115** | **149-153** | **207-207** | **208-218** | **247-247** | **247-251** | **218-223** |
| Brownhill Creek 6 | **205-205** | | **176-180** | **213-215** | **182-190** | **106-106** | **127-131** | **163-167** | **208-218** | **247-258** | **235-251** | **218-220** |
| Brownhill Creek 7 | **195-207** | | **176-180** | **213-215** | **173-173** | **106-112** | **131-149** | **177-195** | **218-218** | **247-268** | **241-245** | **221-225** |
| Brownhill Creek 8 | **201-207** | | **172-178** | **211-215** | **190-190** | **115-115** | **145-145** | **163-167** | **208-276** | **247-267** | **235-241** | **220-225** |
| Brownhill Creek 9 | **207-207** | | **168-176** | **215-225** | **178-190** | **112-115** | **127-143** | **163-195** | **218-276** | **258-267** | **235-251** | **222-223** |
| Brownhill Creek 10 | **195-207** | | **172-176** | **213-215** | **173-190** | **106-112** | **131-145** | **171-177** | **208-218** | **247-247** | **241-247** | **225-225** |
| Brownhill Creek 11 | **207-207** | | **168-172** | **215-215** | **173-190** | **106-115** | **139-143** | **177-195** | **218-276** | **247-247** | **245-247** | **221-221** |
| Brownhill Creek 12 | **207-207** | | **172-178** | **213-215** | **190-190** | **115-115** | **139-149** | **173-207** | **244-276** | **258-268** | **241-241** | **223-225** |
| Brownhill Creek 13 | **195-201** | | **176-176** | **215-215** | **182-190** | **109-112** | **131-145** | **167-173** | **208-208** | **258-268** | **241-241** | **218-220** |
| Brownhill Creek 14 | **207-207** | | **170-176** | **215-233** | **173-190** | **106-108** | **127-145** | **207-207** | **218-276** | **268-271** | **241-245** | **218-225** |
| Brownhill Creek 15 | **195-199** | | **172-182** | **211-213** | **173-184** | **108-109** | **131-139** | **177-211** | **208-276** | **258-271** | **241-251** | **218-221** |
| Brownhill Creek 16 | **203-207** | | **172-178** | **211-211** | **182-184** | **115-115** | **133-143** | **163-207** | **208-208** | **247-268** | **245-251** | **223-223** |
| Brownhill Creek 17 | **195-205** | | **168-182** | **215-215** | **178-190** | **115-124** | **131-139** | **177-211** | **244-276** | **247-258** | **241-251** | **223-225** |
| Brownhill Creek 18 | **199-199** | | **168-180** | **215-215** | **182-190** | **112-115** | **127-133** | **177-177** | **208-276** | **258-268** | **241-247** | **218-225** |
| Brownhill Creek 19 | **207-207** | | **168-176** | **213-215** | **190-190** | **106-124** | **137-143** | **167-173** | **208-276** | **258-258** | **235-247** | **223-223** |
| Brownhill Creek 20 | **199-207** | | **172-172** | **211-211** | **182-190** | **109-112** | **127-141** | **177-177** | **218-276** | **258-258** | **241-247** | **218-225** |
| Brownhill Creek 21 | **199-207** | | **180-180** | **215-215** | **173-190** | **106-115** | **131-145** | **163-195** | **218-276** | **247-247** | **241-247** | **220-225** |
| Brownhill Creek 22 | **207-207** | | **176-176** | **213-215** | **184-190** | **108-112** | **139-139** | **167-195** | **208-276** | **247-268** | **247-251** | **225-225** |
| Brownhill Creek 23 | **195-199** | | **172-176** | **215-215** | **182-182** | **106-115** | **139-139** | **173-177** | **208-276** | **258-268** | **235-241** | **220-225** |
| Brownhill Creek 24 | **201-207** | | **176-182** | **215-215** | **173-182** | **112-115** | **127-131** | **167-167** | **208-208** | **258-268** | **241-241** | **220-225** |
| Brownhill Creek 25 | **199-207** | | **170-172** | **211-213** | **173-178** | **109-115** | **143-149** | **207-207** | **218-218** | **258-268** | **241-247** | **218-225** |
| Brownhill Creek 26 | **199-207** | | **180-182** | **215-215** | **184-190** | **109-112** | **127-133** | **177-211** | **244-276** | **258-268** | **247-247** | **225-225** |

**Table S4.** (continued)

| **Sample code** | **DCA05** | **DCA18** | **Gapu 71A** | **DCA14** | **PA(ATT)2** | **DCA08** | **DCA09** | **DCA01** | **DCA15** | **DCA03** | **EMO3** |
| --- | --- | --- | --- | --- | --- | --- | --- | --- | --- | --- | --- |
| Brownhill Creek 27 | **199-201** | **176-178** | **211-215** | **182-190** | **108-109** | **131-143** | **173-211** | **218-218** | **247-247** | **241-241** | **218-220** |
| Brownhill Creek 28 | **199-207** | **168-168** | **215-215** | **182-182** | **109-115** | **131-145** | **167-211** | **208-244** | **258-268** | **241-247** | **220-225** |
| Brownhill Creek 29 | **199-207** | **180-180** | **211-215** | **173-190** | **112-124** | **139-143** | **167-167** | **218-218** | **247-247** | **241-251** | **220-220** |
| Lonsdale 1 | **205-207** | **172-180** | **215-215** | **190-190** | **108-109** | **137-137** | **167-211** | **244-276** | **258-271** | **235-245** | **222-223** |
| Lonsdale 2 | **199-207** | **170-178** | **215-215** | **182-190** | **109-115** | **145-149** | **177-185** | **218-218** | **271-271** | **241-241** | **218-223** |
| Lonsdale 3 | **205-207** | **176-176** | **215-215** | **190-190** | **109-124** | **139-143** | **167-211** | **208-278** | **247-247** | **235-251** | **222-225** |
| Lonsdale 4 | **199-201** | **170-172** | **215-215** | **190-190** | **115-115** | **131-153** | **173-205** | **208-208** | **247-247** | **235-241** | **220-220** |
| Lonsdale 5 | **203-207** | **170-176** | **213-215** | **173-190** | **106-112** | **131-141** | **163-207** | **208-208** | **258-268** | **235-239** | **223-225** |
| Lonsdale 6 | **199-201** | **176-176** | **215-215** | **190-190** | **109-115** | **133-137** | **167-195** | **218-218** | **247-271** | **241-241** | **220-223** |
| Lonsdale 7 | **199-203** | **168-180** | **213-215** | **173-190** | **115-115** | **131-139** | **177-203** | **218-244** | **247-271** | **241-241** | **220-225** |
| Lonsdale 8 | **207-207** | **176-176** | **211-213** | **182-190** | **109-109** | **131-139** | **163-195** | **208-208** | **268-268** | **235-255** | **220-225** |
| Lonsdale 9 | **203-207** | **170-180** | **213-215** | **182-190** | **109-124** | **139-145** | **167-207** | **208-218** | **247-247** | **241-251** | **218-218** |
| Lonsdale 10 | **199-201** | **172-172** | **211-215** | **173-184** | **109-112** | **143-153** | **167-173** | **218-244** | **247-271** | **241-247** | **218-225** |
| Lonsdale 11 | **199-207** | **176-176** | **215-225** | **149-173** | **112-115** | **131-143** | **167-167** | **208-218** | **247-268** | **241-247** | **220-223** |
| Lonsdale 12 | **199-202** | **176-176** | **215-225** | **173-190** | **106-112** | **131-131** | **167-207** | **218-244** | **258-268** | **241-251** | **223-223** |
| Lonsdale 13 | **199-199** | **176-180** | **215-215** | **173-190** | **106-109** | **139-149** | **167-195** | **218-276** | **247-247** | **239-241** | **220-220** |
| Lonsdale 14 | **207-209** | **170-178** | **215-215** | **173-190** | **115-115** | **127-139** | **167-167** | **218-244** | **258-271** | **235-247** | **218-223** |
| Lonsdale 15 | **199-207** | **170-180** | **213-215** | **182-190** | **112-115** | **137-145** | **167-185** | **218-276** | **247-271** | **235-241** | **220-223** |
| Lonsdale 16* | **201-202** | **176-218** | **215-215** | **184-190** | **115-124** | **143-153** | **163-167** | **218-218** | **247-268** | **247-251** | **213-218** |
| Lonsdale 17 | **205-207** | **178-178** | **215-215** | **190-190** | **106-115** | **137-139** | **177-211** | **244-270** | **247-258** | **235-247** | **223-223** |
| Lonsdale 18 | **207-207** | **176-182** | **211-215** | **182-190** | **115-124** | **133-149** | **177-213** | **208-218** | **247-268** | **241-251** | **218-220** |
| Lonsdale 19 | **201-205** | **176-178** | **211-215** | **172-190** | **109-115** | **127-143** | **167-185** | **218-218** | **247-258** | **235-241** | **222-223** |
| Lonsdale 20 | **199-201** | **168-176** | **211-213** | **173-190** | **106-109** | **133-149** | **177-211** | **208-218** | **247-258** | **241-251** | **218-221** |
| Lonsdale 21 | **207-207** | **172-176** | **215-225** | **149-190** | **112-115** | **137-143** | **167-195** | **218-270** | **247-268** | **235-241** | **220-220** |
| Lonsdale 22* | **201-202** | **170-218** | **211-215** | **173-190** | **115-115** | **133-153** | **173-211** | **208-218** | **247-268** | **233-245** | **213-218** |
| Lonsdale 23 | **199-209** | **176-180** | **213-215** | **190-190** | **109-109** | **137-151** | **199-207** | **218-276** | **247-268** | **235-241** | **218-223** |
| Lonsdale 24 | **195-207** | **176-180** | **213-215** | **182-190** | **112-115** | **131-145** | **183-207** | **208-218** | **247-258** | **241-245** | **222-225** |
| Lonsdale 25 | **199-199** | **168-172** | **215-215** | **182-190** | **106-115** | **127-131** | **207-207** | **208-244** | **247-247** | **235-235** | **218-225** |
| Lonsdale 26 | **207-207** | **172-176** | **213-215** | **173-182** | **106-106** | **149-149** | **163-167** | **208-276** | **247-268** | **241-245** | **221-223** |
| Lonsdale 27 | **199-207** | **180-180** | **215-233** | **173-190** | **115-115** | **133-141** | **163-195** | **208-218** | **247-271** | **235-247** | **220-221** |
| Lonsdale 28 | **199-205** | **178-178** | **215-233** | **190-190** | **106-109** | **131-139** | **173-177** | **208-276** | **247-268** | **241-241** | **220-223** |
| Lonsdale 29 | **203-209** | **176-182** | **215-215** | **190-190** | **109-124** | **139-149** | **167-211** | **244-276** | **247-268** | **235-251** | **218-225** |
| Lonsdale 30 | **199-205** | **168-172** | **211-233** | **173-190** | **112-112** | **133-139** | **167-211** | **218-218** | **247-247** | **241-251** | **220-221** |
| **NSW:** |  |  |  |  |  |  |  |  |  |  |  |
| Camden Park 1* | **202-202** | **168-218** | **213-237** | **182-182** | **106-106** | **127-127** | **167-177** | **238-238** | **258-258** | **233-233** | **212-213** |
| Camden Park 2* | **199-207** | **178-218** | **211-235** | **149-190** | **106-124** | **127-127** | **175-207** | **208-218** | **258-268** | **233-241** | **212-223** |
| Camden Park 3* | **199-199** | **168-170** | **233-233** | **150-182** | **106-106** | **127-127** | **167-175** | **218-238** | **271-271** | **233-241** | **213-221** |
| Camden Park 4* | **199-201** | **170-176** | **215-233** | **148-150** | **106-106** | **127-133** | **175-215** | **214-218** | **247-271** | **241-247** | **213-221** |
| Camden Park 5* | **199-202** | **168-176** | **233-235** | **150-173** | **106-108** | **127-143** | **167-219** | **218-238** | **247-247** | **241-241** | **213-221** |
| Camden Park 6* | **199-202** | **170-180** | **215-233** | **173-182** | **106-108** | **127-143** | **167-219** | **218-218** | **247-247** | **241-247** | **213-221** |
| Camden Park 7* | **199-202** | **168-176** | **215-233** | **150-173** | **106-106** | **127-143** | **167-177** | **218-238** | **247-271** | **233-247** | **220-221** |
| Camden Park 8* | **199-202** | **168-176** | **235-237** | **150-173** | **106-106** | **127-143** | **219-219** | **218-238** | **247-258** | **233-241** | **220-221** |
| Camden Park 9* | **202-202** | **170-218** | **233-235** | **150-150** | **106-106** | **127-133** | **177-177** | **218-276** | **247-271** | **241-241** | **213-221** |
| Camden Park 10* | **200-202** | **180-194** | **233-235** | **149-173** | **106-118** | **125-133** | **167-217** | **214-238** | **247-247** | **233-241** | **213-213** |
| Camden Park 11* | **202-202** | **168-170** | **233-235** | **150-182** | **106-118** | **133-133** | **177-219** | **214-238** | **247-258** | **241-241** | **213-221** |
| Camden Park 12* | **202-202** | **170-188** | **213-213** | **150-150** | **106-106** | **127-143** | **219-219** | **218-238** | **247-258** | **233-241** | **213-221** |
| Camden Park 13* | **199-200** | **168-170** | **213-213** | **149-149** | **106-106** | **127-133** | **175-217** | **218-242** | **247-247** | **233-233** | **212-225** |
| Camden Park 14* | **199-199** | **168-218** | **213-237** | **149-150** | **106-106** | **127-127** | **167-219** | **218-238** | **258-258** | **233-241** | **213-225** |

**Table S4.** (continued)

| **Sample code** | **DCA05** | | **DCA18** | **Gapu 71A** | **DCA14** | **PA(ATT)2** | **DCA08** | **DCA09** | **DCA01** | **DCA15** | **DCA03** | **EMO3** |
| --- | --- | --- | --- | --- | --- | --- | --- | --- | --- | --- | --- | --- |
| Camden Park 15* | | **199-201** | **168-176** | **215-237** | **150-190** | **106-115** | **127-145** | **195-219** | **208-276** | **258-268** | **233-241** | **220-225** |
| Camden Park 16* | | **199-202** | **194-218** | **235-237** | **149-173** | **106-112** | **133-143** | **167-217** | **218-242** | **247-258** | **241-257** | **213-213** |
| Camden Park 17* | **199-202** | | **170-180** | **213-215** | **150-173** | **106-112** | **127-143** | **177-219** | **214-276** | **247-271** | **233-241** | **220-221** |
| Camden Park 18* | **199-200** | | **170-188** | **213-237** | **149-150** | **106-106** | **125-125** | **177-189** | **214-242** | **247-247** | **233-241** | **212-225** |
| Camden Park 19* | **199-202** | | **168-180** | **235-237** | **150-182** | **106-112** | **125-125** | **167-219** | **218-238** | **247-258** | **241-241** | **213-221** |
| Camden Park 20* | **200-202** | | **176-180** | **213-215** | **150-173** | **106-106** | **127-133** | **167-219** | **218-238** | **247-247** | **233-241** | **213-220** |
| Camden Park 21* | **200-202** | | **168-218** | **213-235** | **149-150** | **106-118** | **125-125** | **177-207** | **214-218** | **247-258** | **233-233** | **213-213** |
| Camden Park 22* | **199-200** | | **180-220** | **233-237** | **150-182** | **106-118** | **131-131** | **175-219** | **276-276** | **247-258** | **233-241** | **221-225** |
| Camden Park 23* | **199-202** | | **168-180** | **213-237** | **150-173** | **106-108** | **131-143** | **167-177** | **214-276** | **247-258** | **241-247** | **213-225** |
| Camden Park 24* | **199-200** | | **170-180** | **213-235** | **150-150** | **106-112** | **127-143** | **217-219** | **218-242** | **247-247** | **241-257** | **212-221** |
| Camden Park 25* | **202-202** | | **180-188** | **233-237** | **150-173** | **106-118** | **133-143** | **167-219** | **242-276** | **247-247** | **233-257** | **212-225** |
| Mont Annan 1 | **202-202** | | **170-188** | **237-237** | **149-150** | **106-106** | **125-125** | **175-227** | **214-214** | **247-247** | **233-233** | **211-212** |
| Mont Annan 2* | **199-202** | | **180-202** | **213-237** | **150-150** | **106-112** | **123-135** | **177-219** | **218-242** | **247-247** | **233-233** | **212-213** |
| Mont Annan 3 | **202-202** | | **170-170** | **237-249** | **146-149** | **106-118** | **125-143** | **171-233** | **242-244** | **247-247** | **237-237** | **211-212** |
| Mont Annan 4 | **202-202** | | **182-220** | **233-237** | **147-149** | **106-106** | **123-125** | **169-171** | **236-242** | **247-247** | **233-237** | **212-213** |
| Mont Annan 5 | **202-202** | | **170-202** | **233-233** | **149-150** | **106-106** | **123-125** | **167-219** | **214-214** | **247-247** | **233-237** | **213-213** |
| Mont Annan 6 | **202-202** | | **170-182** | **233-237** | **149-150** | **106-106** | **125-125** | **219-219** | **238-238** | **247-247** | **237-237** | **211-211** |
| Mont Annan 7 | **202-202** | | **170-170** | **237-249** | **146-150** | **106-118** | **123-143** | **219-227** | **242-244** | **247-247** | **237-237** | **211-211** |
| Mont Annan 8* | **201-202** | | **170-180** | **237-237** | **149-150** | **106-106** | **127-127** | **171-219** | **214-276** | **247-247** | **233-237** | **211-212** |
| Mont Annan 9 | **200-202** | | **170-218** | **213-233** | **150-150** | **106-118** | **125-125** | **219-219** | **242-242** | **247-247** | **233-233** | **211-212** |
| Mont Annan 10 | **202-202** | | **170-218** | **237-237** | **149-150** | **106-118** | **123-125** | **171-219** | **214-242** | **247-247** | **233-237** | **212-213** |
| Mont Annan 11 | **202-202** | | **170-182** | **237-237** | **148-150** | **106-106** | **125-125** | **223-227** | **238-238** | **247-247** | **233-237** | **211-212** |
| Mont Annan 12 | **200-202** | | **170-170** | **235-237** | **149-150** | **106-118** | **123-125** | **171-219** | **242-242** | **247-247** | **237-237** | **211-213** |
| Mont Annan 13 | **202-202** | | **182-202** | **213-237** | **149-150** | **106-118** | **123-125** | **195-227** | **214-242** | **247-247** | **233-237** | **208-211** |
| Mont Annan 14 | **202-202** | | **182-218** | **213-237** | **150-150** | **106-118** | **125-125** | **189-195** | **214-242** | **247-247** | **233-237** | **211-211** |
| Mont Annan 15 | **202-202** | | **170-202** | **235-237** | **149-150** | **106-106** | **125-125** | **167-219** | **214-242** | **247-247** | **233-237** | **212-213** |
| Mont Annan 16 | **202-202** | | **170-216** | **217-237** | **150-150** | **106-109** | **125-125** | **167-219** | **214-238** | **247-247** | **233-233** | **211-213** |
| Mont Annan 17* | **202-202** | | **170-170** | **237-237** | **149-150** | **106-118** | **125-127** | **175-219** | **218-238** | **247-258** | **237-241** | **208-225** |
| Mont Annan 18 | **202-202** | | **170-216** | **237-237** | **146-150** | **106-106** | **125-125** | **219-227** | **214-238** | **247-247** | **233-233** | **211-212** |
| Mont Annan 19 | **202-202** | | **170-188** | **219-237** | **149-150** | **106-106** | **125-125** | **227-233** | **214-238** | **247-247** | **233-237** | **211-212** |
| Mont Annan 20 | **200-202** | | **216-216** | **217-237** | **150-150** | **106-106** | **123-125** | **171-195** | **214-242** | **247-247** | **233-237** | **212-212** |
| Mont Annan 21 | **202-202** | | **216-216** | **237-237** | **150-150** | **106-106** | **123-125** | **171-195** | **214-242** | **247-247** | **237-237** | **208-208** |
| Mont Annan 22 | **202-202** | | **170-170** | **235-237** | **149-150** | **106-106** | **123-133** | **169-227** | **236-238** | **247-247** | **233-237** | **212-212** |
| Mont Annan 23 | **200-202** | | **182-216** | **237-237** | **150-150** | **106-118** | **123-123** | **171-195** | **214-242** | **247-247** | **233-233** | **208-212** |
| Mont Annan 24 | **200-202** | | **216-216** | **227-237** | **149-150** | **106-106** | **123-125** | **171-219** | **226-238** | **247-247** | **233-233** | **208-212** |
| Mont Annan 25 | **200-202** | | **216-216** | **237-237** | **149-150** | **106-106** | **125-125** | **195-227** | **242-242** | **247-247** | **233-237** | **212-212** |
| Bringelly 1 | **202-202** | | **202-188** | **233-237** | **150-150** | **106-118** | **125-125** | **169-175** | **214-242** | **247-247** | **233-233** | **213-213** |
| Bringelly 2 | **200-202** | | **202-218** | **237-237** | **150-150** | **106-118** | **125-125** | **167-195** | **214-260** | **247-247** | **233-233** | **208-213** |
| Bringelly 3 | **202-202** | | **202-214** | **213-237** | **150-150** | **106-118** | **125-125** | **167-169** | **236-242** | **247-247** | **233-233** | **208-213** |
| Bringelly 4 | **200-202** | | **202-218** | **213-237** | **149-150** | **109-118** | **125-125** | **169-219** | **236-238** | **247-247** | **237-237** | **208-212** |
| Bringelly 5 | **202-202** | | **170-218** | **233-235** | **146-150** | **118-118** | **125-125** | **171-219** | **236-242** | **247-247** | **233-233** | **211-213** |
| Bringelly 6 | **202-202** | | **202-218** | **213-237** | **149-150** | **106-118** | **125-125** | **169-175** | **214-238** | **247-247** | **233-237** | **212-213** |
| Bringelly 7 | **200-202** | | **202-202** | **237-237** | **149-149** | **106-118** | **125-125** | **171-175** | **236-242** | **247-247** | **233-237** | **208-213** |
| Bringelly 8 | **200-200** | | **212-212** | **217-223** | **145-148** | **118-118** | **125-125** | **219-219** | **238-242** | **247-247** | **233-237** | **213-213** |
| Bringelly 9 | **200-202** | | **202-218** | **217-237** | **148-150** | **106-106** | **125-125** | **195-219** | **238-238** | **247-247** | **233-237** | **208-213** |
| Bringelly 10 | **202-202** | | **170-202** | **237-237** | **149-149** | **106-106** | **125-125** | **169-175** | **214-242** | **247-247** | **233-233** | **213-213** |
| Bringelly 11 | **200-202** | | **202-216** | **213-217** | **149-150** | **106-106** | **125-125** | **167-175** | **214-214** | **247-247** | **233-233** | **211-213** |
| Bringelly 12 | **202-202** | | **218-218** | **235-237** | **148-150** | **106-109** | **125-125** | **167-167** | **238-260** | **247-247** | **233-233** | **208-213** |

**Table S4.** (continued)

| **Sample code** | **DCA05** | **DCA18** | **Gapu 71A** | **DCA14** | **PA(ATT)2** | **DCA08** | **DCA09** | **DCA01** | **DCA15** | **DCA03** | **EMO3** |
| --- | --- | --- | --- | --- | --- | --- | --- | --- | --- | --- | --- |
| Bringelly 13 | **202-202** | **216-218** | **235-237** | **148-149** | **109-118** | **125-125** | **195-229** | **238-242** | **247-247** | **233-233** | **208-208** |
| Bringelly 14 | **202-202** | **188-216** | **213-237** | **150-150** | **106-106** | **125-125** | **219-219** | **214-242** | **247-247** | **233-233** | **213-213** |
| Bringelly 15 | **200-202** | **202-202** | **235-237** | **150-150** | **106-106** | **125-125** | **169-175** | **238-242** | **247-247** | **233-233** | **211-213** |
| Bringelly 16 | **200-202** | **216-218** | **213-233** | **149-150** | **106-118** | **125-125** | **175-219** | **226-236** | **247-247** | **233-233** | **212-213** |
| Bringelly 17 | **202-202** | **202-218** | **213-213** | **148-150** | **109-118** | **125-143** | **169-227** | **238-242** | **247-247** | **233-237** | **212-213** |
| Bringelly 18 | **202-202** | **164-184** | **213-213** | **146-149** | **118-118** | **123-125** | **167-175** | **238-260** | **247-247** | **233-233** | **212-213** |
| Bringelly 19 | **202-202** | **164-218** | **237-237** | **148-149** | **106-106** | **125-125** | **169-175** | **214-242** | **247-247** | **233-237** | **212-213** |
| Bringelly 20 | **202-202** | **170-202** | **233-237** | **146-148** | **106-106** | **125-125** | **171-219** | **214-238** | **247-247** | **233-233** | **213-213** |
| Bringelly 21* | **207-207** | **172-202** | **211-215** | **150-190** | **106-106** | **127-139** | **207-219** | **242-242** | **247-247** | **233-253** | **213-223** |
| Bringelly 22 | **200-202** | **194-202** | **237-249** | **146-150** | **106-118** | **125-125** | **175-175** | **214-242** | **247-247** | **233-233** | **212-213** |
| Bringelly 23 | **200-200** | **170-202** | **237-237** | **149-149** | **118-118** | **125-125** | **169-205** | **236-238** | **247-247** | **233-233** | **212-213** |
| Bringelly 24 | **202-202** | **216-218** | **213-237** | **146-148** | **106-118** | **125-125** | **189-195** | **214-242** | **247-247** | **233-237** | **213-213** |
| Bringelly 25 | **200-202** | **164-218** | **213-237** | **148-149** | **106-118** | **123-125** | **167-169** | **242-242** | **247-247** | **233-237** | **212-213** |
| Bringelly 26 | **200-202** | **164-202** | **237-237** | **149-149** | **106-106** | **125-125** | **175-219** | **214-242** | **247-247** | **233-233** | **213-213** |
| Bringelly 27 | **202-202** | **216-218** | **235-237** | **148-149** | **106-109** | **125-125** | **167-229** | **214-238** | **247-247** | **237-237** | **213-213** |
| Bringelly 28 | **202-202** | **182-216** | **217-237** | **148-150** | **106-118** | **125-141** | **169-195** | **214-214** | **247-247** | **237-237** | **211-213** |
| Bringelly 29 | **200-202** | **202-216** | **213-237** | **149-150** | **106-118** | **125-125** | **219-219** | **214-214** | **247-247** | **237-237** | **213-213** |
| Luddenham 1 | **202-202** | **182-218** | **235-235** | **150-150** | **106-118** | **125-125** | **219-237** | **214-244** | **247-247** | **233-233** | **213-213** |
| Luddenham 2 | **202-202** | **202-216** | **233-235** | **149-149** | **106-106** | **125-125** | **211-227** | **214-242** | **247-247** | **233-237** | **208-213** |
| Luddenham 3 | **202-202** | **170-202** | **235-237** | **149-149** | **106-118** | **125-125** | **167-189** | **214-238** | **247-247** | **233-233** | **212-213** |
| Luddenham 4 | **202-202** | **202-202** | **233-237** | **149-150** | **106-118** | **125-125** | **167-167** | **242-242** | **247-247** | **233-233** | **213-213** |
| Luddenham 5 | **200-202** | **202-216** | **237-237** | **149-150** | **106-118** | **123-125** | **219-219** | **238-242** | **247-247** | **233-233** | **208-212** |
| Luddenham 6 | **202-202** | **202-216** | **233-235** | **149-150** | **106-118** | **125-141** | **189-195** | **214-242** | **247-247** | **233-233** | **212-213** |
| Luddenham 7 | **202-202** | **170-188** | **233-237** | **149-150** | **106-118** | **125-125** | **167-237** | **214-238** | **247-247** | **233-233** | **212-213** |
| Luddenham 8 | **200-202** | **170-218** | **217-237** | **149-150** | **106-118** | **125-125** | **167-167** | **214-242** | **247-247** | **233-233** | **213-213** |
| Luddenham 9 | **202-202** | **170-216** | **237-237** | **149-150** | **106-106** | **125-125** | **227-237** | **214-238** | **247-247** | **233-237** | **212-213** |
| Luddenham 10 | **202-202** | **170-170** | **217-235** | **148-150** | **106-118** | **125-125** | **167-227** | **214-242** | **247-247** | **233-233** | **212-213** |
| Luddenham 11 | **200-202** | **202-214** | **213-233** | **149-150** | **106-106** | **125-125** | **167-219** | **242-242** | **247-247** | **233-233** | **213-213** |
| Luddenham 12 | **202-202** | **170-216** | **233-233** | **146-149** | **106-106** | **125-133** | **219-223** | **214-238** | **247-247** | **233-233** | **211-213** |
| Luddenham 13 | **202-202** | **188-216** | **233-235** | **150-150** | **106-106** | **125-141** | **167-167** | **214-214** | **247-247** | **233-233** | **208-213** |
| Luddenham 14 | **202-202** | **202-202** | **217-235** | **150-150** | **100-106** | **125-125** | **171-195** | **242-260** | **247-247** | **233-233** | **212-213** |
| Luddenham 15 | **202-202** | **182-214** | **233-235** | **150-150** | **106-109** | **125-125** | **175-219** | **214-226** | **247-247** | **233-233** | **208-212** |
| Luddenham 16 | **200-202** | **170-202** | **233-237** | **148-149** | **106-106** | **125-133** | **167-167** | **242-242** | **247-247** | **233-233** | **213-213** |
| Luddenham 17 | **202-202** | **182-216** | **213-217** | **149-149** | **106-118** | **123-125** | **189-219** | **242-242** | **247-247** | **233-233** | **208-213** |
| Luddenham 18 | **200-202** | **202-218** | **213-233** | **149-150** | **106-118** | **125-125** | **195-213** | **238-260** | **247-247** | **233-233** | **213-213** |
| Luddenham 19 | **202-202** | **170-170** | **213-237** | **146-149** | **118-118** | **125-135** | **167-219** | **214-214** | **247-247** | **233-233** | **213-213** |
| Luddenham 20 | **200-202** | **170-218** | **217-237** | **149-150** | **106-118** | **125-125** | **219-219** | **214-238** | **247-247** | **233-233** | **211-213** |
| Luddenham 21 | **202-202** | **170-188** | **217-233** | **149-150** | **106-106** | **125-125** | **167-219** | **214-238** | **247-247** | **233-233** | **213-213** |
| Luddenham 22 | **202-202** | **170-170** | **217-237** | **146-149** | **106-106** | **125-135** | **219-219** | **214-242** | **247-247** | **233-237** | **213-213** |
| Luddenham 23 | **200-202** | **202-216** | **235-237** | **149-150** | **106-106** | **125-125** | **167-167** | **214-226** | **247-247** | **233-233** | **213-213** |
| Luddenham 24 | **202-202** | **218-218** | **217-235** | **148-149** | **106-118** | **125-135** | **167-171** | **214-242** | **247-247** | **233-233** | **212-213** |
| Luddenham 25 | **202-202** | **188-202** | **233-233** | **150-150** | **106-118** | **125-125** | **167-195** | **214-242** | **247-247** | **233-233** | **213-213** |
| Maitland Park 1 | **202-202** | **184-196** | **213-227** | **146-146** | **106-106** | **123-125** | **169-175** | **214-236** | **247-247** | **233-233** | **213-213** |
| Maitland Park 2 | **202-202** | **176-218** | **223-227** | **146-146** | **106-109** | **125-125** | **169-169** | **236-244** | **247-247** | **233-237** | **212-212** |
| Maitland Park 3 | **200-202** | **164-206** | **223-223** | **146-150** | **106-118** | **125-125** | **169-169** | **238-244** | **247-247** | **233-233** | **207-212** |
| Maitland Park 4 | **202-202** | **186-202** | **213-227** | **146-150** | **106-109** | **123-123** | **169-171** | **242-244** | **247-247** | **233-233** | **207-212** |
| Maitland Park 5 | **202-202** | **164-186** | **233-233** | **146-150** | **106-106** | **125-133** | **169-169** | **236-236** | **247-247** | **233-233** | **212-212** |
| Harpers Hill 1 | **202-202** | **170-218** | **233-237** | **149-149** | **106-106** | **125-125** | **169-169** | **236-236** | **247-247** | **233-233** | **212-212** |

**Table S4.** (continued)

| **Sample code** | **DCA05** | **DCA18** | **Gapu 71A** | **DCA14** | **PA(ATT)2** | **DCA08** | **DCA09** | **DCA01** | **DCA15** | **DCA03** | **EMO3** |
| --- | --- | --- | --- | --- | --- | --- | --- | --- | --- | --- | --- |
| Harpers Hill 2 | **200-202** | **164-218** | **237-237** | **147-149** | **106-106** | **123-135** | **175-175** | **214-244** | **247-247** | **233-233** | **212-212** |
| Harpers Hill 3 | **200-202** | **170-218** | **233-235** | **149-150** | **106-106** | **125-135** | **219-233** | **242-242** | **247-247** | **233-233** | **211-213** |
| Harpers Hill 4 | **202-202** | **170-188** | **213-233** | **149-150** | **106-106** | **125-135** | **169-219** | **236-240** | **247-247** | **233-233** | **212-213** |
| Harpers Hill 5 | **200-202** | **196-218** | **227-233** | **146-147** | **106-118** | **135-141** | **169-219** | **226-242** | **247-247** | **233-233** | **207-212** |
| Harpers Hill 6 | **202-202** | **164-164** | **233-237** | **148-150** | **106-106** | **125-133** | **169-219** | **226-236** | **247-247** | **233-233** | **212-212** |
| Harpers Hill 7 | **202-202** | **194-218** | **213-237** | **146-150** | **106-106** | **125-135** | **169-175** | **214-236** | **247-247** | **233-233** | **212-213** |
| Harpers Hill 8 | **202-202** | **170-170** | **227-233** | **147-149** | **106-106** | **125-135** | **169-175** | **242-244** | **247-247** | **233-233** | **212-213** |
| Harpers Hill 9 | **200-200** | **164-176** | **237-237** | **146-150** | **106-106** | **125-133** | **169-219** | **226-238** | **247-247** | **233-233** | **212-212** |
| Harpers Hill 10 | **200-202** | **198-218** | **223-235** | **149-150** | **106-118** | **123-125** | **169-233** | **236-244** | **247-247** | **233-233** | **213-213** |
| Harpers Hill 11 | **202-202** | **164-218** | **233-235** | **149-150** | **106-106** | **125-125** | **169-175** | **242-242** | **247-247** | **233-233** | **212-212** |
| Harpers Hill 12 | **202-202** | **196-218** | **227-237** | **147-150** | **106-106** | **125-133** | **169-175** | **236-238** | **247-247** | **233-233** | **207-212** |
| Harpers Hill 13 | **200-202** | **188-188** | **213-235** | **146-150** | **118-118** | **125-133** | **189-189** | **208-242** | **247-247** | **233-237** | **211-212** |
| Harpers Hill 14 | **202-202** | **170-218** | **237-237** | **147-149** | **106-106** | **125-125** | **169-189** | **236-242** | **247-247** | **233-233** | **212-212** |
| Harpers Hill 15 | **202-202** | **194-194** | **227-233** | **147-150** | **106-106** | **123-125** | **169-175** | **236-242** | **247-247** | **233-237** | **212-213** |
| Harpers Hill 16 | **200-202** | **218-218** | **233-233** | **149-150** | **106-118** | **125-141** | **175-189** | **238-242** | **247-247** | **233-233** | **207-212** |
| Harpers Hill 17 | **200-202** | **170-196** | **227-237** | **149-150** | **106-118** | **125-125** | **169-175** | **214-236** | **247-247** | **233-233** | **213-213** |
| Harpers Hill 18 | **200-202** | **190-216** | **213-249** | **146-150** | **106-106** | **123-125** | **169-219** | **242-244** | **247-247** | **233-233** | **212-212** |
| Harpers Hill 19 | **200-202** | **164-218** | **213-235** | **146-150** | **106-106** | **135-135** | **169-219** | **236-236** | **247-247** | **233-233** | **212-213** |
| Harpers Hill 20 | **202-202** | **196-218** | **233-235** | **149-150** | **106-118** | **125-135** | **169-233** | **214-236** | **247-247** | **233-233** | **212-212** |
| Harpers Hill 21 | **202-202** | **196-218** | **235-235** | **146-150** | **106-118** | **133-135** | **169-169** | **236-242** | **247-247** | **233-233** | **212-213** |
| Harpers Hill 22 | **202-202** | **196-218** | **237-237** | **146-150** | **106-106** | **125-135** | **189-219** | **238-244** | **247-247** | **233-233** | **212-213** |
| Harpers Hill 23 | **202-202** | **170-218** | **213-213** | **149-150** | **106-106** | **123-125** | **189-233** | **238-242** | **247-247** | **233-233** | **212-213** |
| Harpers Hill 24 | **202-202** | **170-184** | **235-237** | **146-146** | **106-106** | **123-125** | **169-169** | **236-242** | **247-247** | **233-233** | **212-212** |
| **Hawaii :** |  |  |  |  |  |  |  |  |  |  |  |
| Maui 1 | **202-202** | **170-186** | **213-213** | **146-146** | **106-118** | **125-133** | **167-167** | **214-214** | **247-247** | **233-237** | **211-213** |
| Maui 2 | **202-202** | **170-186** | **213-249** | **146-149** | **106-118** | **125-125** | **167-167** | **214-214** | **247-247** | **233-233** | **211-213** |
| Maui 3 | **202-202** | **170-170** | **213-213** | **146-146** | **118-118** | **125-125** | **167-169** | **214-214** | **247-247** | **233-237** | **211-213** |
| Maui 4 | **202-202** | **170-186** | **213-213** | **146-149** | **106-118** | **125-133** | **167-169** | **214-242** | **247-247** | **233-233** | **211-212** |
| Maui 5 | **202-202** | **186-186** | **213-249** | **146-146** | **118-118** | **125-133** | **167-167** | **214-214** | **247-247** | **233-237** | **213-213** |
| Maui 6 | **202-202** | **170-186** | **213-213** | **146-149** | **118-118** | **125-125** | **169-169** | **214-238** | **247-247** | **233-237** | **213-213** |
| Maui 7 | **202-202** | **170-170** | **213-249** | **146-146** | **118-118** | **125-125** | **167-167** | **214-214** | **247-247** | **233-237** | **213-213** |
| Maui 8 | **202-202** | **170-186** | **213-249** | **146-146** | **106-118** | **125-133** | **167-167** | **214-214** | **247-247** | **233-233** | **213-213** |
| Maui 9 | **202-202** | **186-186** | **213-249** | **146-146** | **118-118** | **125-133** | **167-167** | **214-214** | **247-247** | **233-237** | **213-213** |
| Maui 10 | **202-202** | **170-170** | **213-213** | **146-146** | **106-118** | **125-133** | **167-169** | **214-238** | **247-247** | **233-237** | **213-213** |
| Maui 11 | **200-202** | **170-170** | **237-249** | **146-149** | **118-118** | **125-125** | **167-169** | **214-238** | **247-247** | **233-233** | **212-213** |
| Maui 12 | **202-202** | **170-186** | **213-213** | **146-149** | **118-118** | **125-133** | **169-169** | **214-238** | **247-247** | **233-237** | **213-213** |
| Maui 13 | **202-202** | **170-186** | **213-213** | **146-149** | **118-118** | **125-125** | **167-169** | **214-214** | **247-247** | **233-233** | **211-213** |
| Maui 14 | **202-202** | **170-186** | **213-213** | **146-149** | **106-118** | **125-133** | **167-169** | **214-238** | **247-247** | **233-237** | **211-211** |
| Maui 15 | **200-202** | **170-170** | **213-213** | **146-149** | **106-118** | **125-125** | **167-169** | **214-214** | **247-247** | **233-237** | **211-213** |
| Maui 16 | **202-202** | **170-170** | **213-213** | **146-149** | **106-118** | **125-133** | **167-169** | **214-238** | **247-247** | **237-237** | **211-211** |
| Maui 17 | **202-202** | **186-186** | **213-249** | **146-149** | **106-118** | **125-125** | **169-169** | **238-242** | **247-247** | **233-233** | **211-211** |
| Maui 18 | **200-202** | **170-170** | **213-249** | **146-149** | **106-118** | **125-133** | **167-167** | **214-238** | **247-247** | **233-237** | **211-211** |
| Maui 19 | **202-202** | **170-186** | **213-213** | **146-149** | **106-118** | **125-133** | **167-169** | **214-238** | **247-247** | **233-237** | **211-211** |
| Maui 20 | **202-202** | **186-186** | **213-213** | **146-149** | **118-118** | **125-133** | **169-169** | **214-238** | **247-247** | **233-237** | **213-213** |
| Maui 21 | **202-202** | **186-186** | **213-249** | **146-146** | **106-118** | **125-133** | **167-169** | **214-238** | **247-247** | **233-233** | **213-213** |
| Maui 22 | **200-202** | **170-186** | **213-249** | **146-146** | **106-106** | **125-133** | **167-169** | **214-238** | **247-247** | **233-233** | **213-213** |
| Maui 23 | **202-202** | **170-186** | **213-213** | **146-149** | **118-118** | **125-125** | **169-169** | **214-238** | **247-247** | **233-237** | **213-213** |
| Maui 24 | **202-202** | **170-186** | **213-213** | **146-146** | **106-118** | **125-133** | **167-167** | **214-214** | **247-247** | **233-237** | **211-213** |
| Maui 25 | **202-202** | **170-186** | **213-213** | **146-149** | **118-118** | **125-125** | **169-169** | **238-238** | **247-247** | **237-237** | **211-213** |

**Table S4.** (continued)

| **Sample code** | **DCA05** | **DCA18** | **Gapu 71A** | **DCA14** | **PA(ATT)2** | **DCA08** | **DCA09** | **DCA01** | **DCA15** | **DCA03** | **EMO3** |
| --- | --- | --- | --- | --- | --- | --- | --- | --- | --- | --- | --- |
| Maui 26 | **202-202** | **170-186** | **213-213** | **146-149** | **118-118** | **125-125** | **167-167** | **214-214** | **247-247** | **233-237** | **211-213** |
| **Cultivars:** |  |  |  |  |  |  |  |  |  |  |  |
| Kaissy | **207-207** | **176-184** | **211-215** | **173-178** | **115-115** | **133-139** | **163-195** | **208-208** | **247-268** | **234-234** | **218-222** |
| Zaity | **207-207** | **174-182** | **215-215** | **178-180** | **115-115** | **129-141** | **189-195** | **208-208** | **247-267** | **251-255** | **220-221** |
| Abbadi | **205-207** | **176-176** | **215-215** | **190-190** | **115-124** | **133-141** | **173-195** | **208-244** | **247-268** | **247-253** | **219-220** |
| Chalchali | **207-207** | **176-184** | **211-215** | **173-178** | **115-115** | **133-139** | **163-195** | **208-208** | **247-268** | **234-234** | **218-222** |
| Chami | **207-209** | **170-176** | **215-215** | **178-190** | **112-115** | **133-133** | **195-205** | **208-216** | **247-247** | **234-247** | **220-222** |
| Souri | **207-207** | **176-176** | **211-215** | **185-190** | **109-115** | **133-137** | **173-195** | **208-216** | **247-268** | **247-251** | **222-222** |
| Merhavia | **207-207** | **170-176** | **215-215** | **188-190** | **112-124** | **137-139** | **195-195** | **208-216** | **247-268** | **245-255** | **220-222** |
| Nabali | **207-207** | **172-176** | **215-215** | **190-190** | **109-115** | **139-143** | **185-195** | **208-218** | **247-258** | **241-251** | **220-222** |
| Hamed | **207-207** | **172-180** | **215-215** | **180-190** | **115-124** | **139-141** | **163-207** | **208-216** | **258-268** | **247-255** | **218-222** |
| Wardan | **201-207** | **174-176** | **213-215** | **172-174** | **106-124** | **129-157** | **173-173** | **208-216** | **247-268** | **234-234** | **220-223** |
| Ladolia | **207-207** | **176-176** | **211-215** | **185-190** | **109-115** | **133-137** | **173-195** | **208-216** | **247-268** | **247-251** | **222-222** |
| Belluti | **201-205** | **166-178** | **213-215** | **174-190** | **109-109** | **133-147** | **177-205** | **208-210** | **247-271** | **234-239** | **222-223** |
| Vallanolia | **207-209** | **170-172** | **215-215** | **178-190** | **112-115** | **133-141** | **195-195** | **208-216** | **247-247** | **247-251** | **220-223** |
| Trylia | **207-207** | **170-176** | **215-215** | **178-190** | **109-115** | **139-141** | **195-205** | **208-216** | **247-268** | **245-251** | **218-220** |
| Uslu | **205-207** | **172-176** | **211-215** | **178-180** | **109-115** | **133-139** | **163-205** | **208-208** | **247-268** | **255-255** | **218-219** |
| Ayvalik | **195-209** | **170-178** | **215-215** | **178-190** | **115-115** | **133-141** | **195-207** | **208-216** | **247-268** | **245-247** | **220-222** |
| Memecik | **207-207** | **170-176** | **215-215** | **180-190** | **112-124** | **139-141** | **195-195** | **208-208** | **247-268** | **247-251** | **218-223** |
| Mastoidis | **195-207** | **162-170** | **215-215** | **178-182** | **106-115** | **141-149** | **183-207** | **208-208** | **265-268** | **234-245** | **222-222** |
| Mirtolia | **195-195** | **170-172** | **215-215** | **178-190** | **112-115** | **133-149** | **183-209** | **216-233** | **268-268** | **241-245** | **222-223** |
| Kalamon | **207-207** | **184-186** | **215-215** | **180-182** | **109-115** | **137-139** | **167-195** | **208-208** | **266-268** | **234-255** | **218-226** |
| Konservolia | **207-207** | **170-184** | **211-215** | **180-190** | **112-124** | **137-139** | **173-199** | **208-208** | **247-268** | **234-255** | **220-222** |
| Megaritiki | **207-215** | **176-178** | **211-215** | **175-178** | **106-115** | **141-155** | **167-173** | **208-216** | **268-271** | **234-246** | **216-222** |
| Lastovka | **207-207** | **168-180** | **211-215** | **186-190** | **115-115** | **133-141** | **195-207** | **208-218** | **247-271** | **234-245** | **218-222** |
| Moraiolo | **207-207** | **178-184** | **215-215** | **173-190** | **112-115** | **141-143** | **185-207** | **216-218** | **247-258** | **234-245** | **223-226** |
| Nocellara Belica | **207-207** | **172-176** | **215-215** | **178-190** | **109-115** | **139-141** | **163-173** | **208-216** | **247-247** | **245-251** | **218-223** |
| Frantoio | **199-207** | **176-178** | **215-225** | **182-190** | **109-115** | **137-143** | **183-207** | **208-272** | **247-268** | **239-245** | **223-223** |
| Giaraffa | **207-207** | **176-184** | **211-215** | **180-190** | **109-124** | **139-139** | **163-195** | **208-208** | **247-268** | **251-255** | **218-220** |
| Pizz'e carroga | **207-207** | **170-182** | **215-215** | **180-190** | **112-124** | **139-141** | **163-173** | **208-208** | **247-268** | **245-251** | **218-222** |
| Carolea | **195-207** | **178-180** | **215-215** | **178-190** | **115-124** | **137-139** | **163-199** | **208-208** | **247-247** | **234-255** | **220-222** |
| Nocellara | **207-207** | **170-176** | **215-215** | **190-190** | **112-124** | **133-139** | **173-195** | **208-216** | **247-268** | **245-251** | **220-222** |
| Itrana | **199-207** | **172-180** | **211-215** | **178-190** | **109-115** | **139-145** | **183-195** | **208-218** | **247-247** | **241-251** | **220-222** |
| Bosana | **203-207** | **178-184** | **213-215** | **190-190** | **112-115** | **133-137** | **173-187** | **208-216** | **258-268** | **245-247** | **222-223** |
| Confetto | **207-209** | **178-182** | **211-215** | **180-190** | **109-115** | **137-139** | **163-173** | **208-208** | **247-268** | **245-255** | **218-223** |
| Picholine | **203-207** | **170-180** | **215-215** | **190-190** | **112-124** | **139-141** | **195-195** | **208-268** | **247-268** | **234-255** | **220-222** |
| Cailletier | **199-207** | **176-178** | **215-225** | **180-190** | **109-115** | **137-139** | **183-207** | **208-270** | **247-247** | **239-245** | **222-223** |
| Olivière | **207-207** | **176-178** | **211-215** | **178-190** | **112-124** | **139-141** | **187-195** | **208-218** | **247-247** | **234-251** | **220-222** |
| Zinzala | **199-207** | **176-184** | **215-243** | **182-190** | **112-112** | **141-159** | **183-185** | **216-220** | **247-268** | **241-245** | **213-223** |
| Capanacce | **199-199** | **172-178** | **215-241** | **182-190** | **112-115** | **137-143** | **173-207** | **208-270** | **247-271** | **239-241** | **223-225** |
| Sabina | **201-207** | **174-186** | **213-215** | **178-186** | **106-109** | **141-141** | **183-196** | **218-229** | **247-268** | **234-234** | **221-223** |
| Galega | **207-215** | **172-180** | **215-229** | **190-190** | **109-109** | **127-139** | **193-195** | **208-233** | **247-271** | **241-255** | **216-218** |
| Empeltre | **199-207** | **170-180** | **213-215** | **178-190** | **112-112** | **133-137** | **187-207** | **216-218** | **247-268** | **245-247** | **216-220** |
| Lechin Granada | **207-207** | **170-172** | **215-215** | **190-190** | **106-115** | **141-143** | **195-207** | **216-218** | **258-268** | **241-247** | **222-226** |
| Picudo | **207-207** | **172-176** | **215-215** | **190-190** | **115-124** | **139-141** | **185-195** | **208-216** | **268-268** | **247-255** | **220-226** |
| Changlot Real | **207-207** | **170-180** | **215-215** | **180-190** | **109-115** | **133-141** | **187-207** | **216-218** | **258-268** | **241-257** | **218-222** |
| Blanqueta | **207-207** | **180-180** | **211-215** | **182-190** | **115-115** | **127-137** | **181-207** | **208-216** | **247-268** | **232-241** | **222-223** |
| Villalonga | **207-207** | **176-180** | **215-215** | **182-190** | **124-124** | **133-149** | **163-183** | **208-216** | **247-268** | **255-257** | **220-222** |
| Arbequina | **203-207** | **168-178** | **215-215** | **190-190** | **115-115** | **141-141** | **185-207** | **208-218** | **247-268** | **234-245** | **222-222** |

**Table S4.** (end)

| **Sample code** | **DCA05** | **DCA18** | **Gapu 71A** | **DCA14** | **PA(ATT)2** | **DCA08** | **DCA09** | **DCA01** | **DCA15** | **DCA03** | **EMO3** |
| --- | --- | --- | --- | --- | --- | --- | --- | --- | --- | --- | --- |
| Gordal Sevillana | **207-207** | **176-180** | **211-215** | **180-190** | **109-124** | **139-139** | **163-195** | **208-208** | **247-268** | **251-255** | **218-220** |
| Picual | **207-207** | **170-176** | **215-215** | **190-190** | **106-124** | **139-143** | **185-193** | **208-218** | **247-258** | **241-251** | **218-226** |
| Cornicabra | **207-207** | **172-180** | **215-215** | **190-190** | **106-124** | **139-145** | **185-195** | **208-218** | **268-268** | **241-251** | **218-222** |
| Hojibianca | **207-207** | **172-180** | **211-215** | **190-190** | **109-115** | **127-139** | **195-207** | **208-218** | **247-258** | **241-251** | **218-222** |
| Lechin de Sevilla | **203-207** | **168-176** | **211-211** | **172-190** | **106-109** | **139-143** | **163-205** | **208-218** | **247-258** | **247-251** | **218-222** |
| Verdial Huevar | **207-207** | **172-180** | **211-215** | **180-190** | **109-115** | **139-143** | **185-195** | **208-218** | **268-268** | **241-251** | **220-226** |
| Verdial de Velez | **207-215** | **172-176** | **213-215** | **182-190** | **109-112** | **127-139** | **177-195** | **208-208** | **247-268** | **245-247** | **222-226** |
| Manzanilla Almeria | **207-207** | **170-180** | **211-215** | **180-190** | **109-115** | **139-145** | **195-207** | **208-218** | **247-268** | **247-255** | **218-222** |
| Negrillo Carlota | **207-207** | **170-176** | **215-215** | **180-190** | **106-109** | **139-145** | **195-207** | **208-216** | **258-268** | **241-251** | **220-226** |
| Pico Limon | **207-207** | **172-176** | **211-215** | **190-190** | **106-124** | **139-141** | **185-195** | **208-218** | **247-258** | **247-255** | **218-226** |
| Manz. Cacerana | **207-207** | **172-176** | **211-215** | **180-190** | **109-115** | **139-141** | **163-185** | **208-218** | **258-268** | **241-255** | **218-222** |
| Manzanilla Sevilla | **207-207** | **172-180** | **215-215** | **180-190** | **115-124** | **139-141** | **163-207** | **208-216** | **258-268** | **247-255** | **218-222** |
| Taksrit | **207-213** | **176-176** | **215-235** | **182-190** | **108-115** | **127-133** | **173-185** | **216-218** | **268-268** | **234-247** | **221-222** |
| Chemlal | **203-207** | **174-174** | **215-233** | **170-180** | **109-112** | **127-139** | **173-177** | **216-218** | **258-268** | **241-255** | **221-222** |
| Zarrazzi | **203-207** | **172-174** | **215-215** | **178-190** | **106-115** | **139-157** | **163-195** | **216-224** | **247-258** | **234-245** | **223-225** |
| Zalmati | **195-201** | **174-178** | **213-215** | **172-190** | **106-115** | **129-137** | **173-173** | **216-220** | **268-268** | **234-241** | **216-223** |
| Meski | **207-209** | **174-176** | **215-215** | **178-190** | **109-112** | **133-139** | **187-195** | **208-208** | **247-247** | **241-245** | **218-222** |
| Chemchali-Jemri | **195-207** | **176-178** | **215-229** | **172-178** | **112-115** | **133-141** | **173-196** | **216-229** | **247-271** | **234-245** | **222-225** |
| Chétoui | **195-207** | **174-178** | **211-229** | **172-190** | **109-112** | **135-139** | **195-195** | **208-208** | **247-247** | **234-241** | **218-220** |
| Chemlali | **195-207** | **174-178** | **211-229** | **172-190** | **109-112** | **135-139** | **195-195** | **208-208** | **247-247** | **234-241** | **218-220** |
| Picholine Maroc. | **207-207** | **172-180** | **215-215** | **180-190** | **106-124** | **139-141** | **195-207** | **208-216** | **247-258** | **241-255** | **220-222** |
| **Cape Town:** |  |  |  |  |  |  |  |  |  |  |  |
| Cape Town 1 | **202-202** | **190-204** | **213-217** | **149-149** | **100-106** | **123-131** | **167-215** | **224-242** | **247-247** | **233-233** | **202-208** |
| Cape Town 2 | **196-200** | **186-202** | **223-255** | **152-152** | **106-115** | **149-169** | **167-167** | **252-272** | **247-247** | **233-279** | **200-212** |
| Cape Town 3 | **196-200** | **186-218** | **225-255** | **147-152** | **100-106** | **123-123** | **167-197** | **238-250** | **247-247** | **233-279** | **200-212** |
| Cape Town 4 | **200-200** | **186-202** | **209-225** | **147-148** | **106-106** | **123-151** | **167-197** | **238-252** | **247-247** | **233-279** | **200-200** |
| Cape Town 5 | **200-200** | **160-186** | **209-223** | **149-152** | **100-106** | **119-139** | **167-169** | **244-250** | **247-247** | **233-235** | **209-212** |
| Cape Town 6 | **202-202** | **164-194** | **219-237** | **146-147** | **106-106** | **131-143** | **169-195** | **214-236** | **247-247** | **233-271** | **200-208** |
| Cape Town 7 | **200-202** | **170-204** | **217-225** | **149-149** | **106-109** | **123-131** | **175-203** | **226-242** | **247-247** | **233-233** | **208-213** |
| Cape Town 8 | **200-202** | **188-204** | **209-211** | **149-152** | **106-106** | **139-157** | **167-199** | **230-250** | **247-247** | **231-233** | **210-211** |
| Cape Town 9 | **200-202** | **164-164** | **217-217** | **149-149** | **106-106** | **127-173** | **169-171** | **214-250** | **247-247** | **233-233** | **201-208** |
| Cape Town 10 | **200-202** | **172-184** | **215-225** | **148-149** | **100-106** | **127-137** | **169-173** | **214-248** | **247-247** | **233-233** | **209-211** |
| Cape Town 11 | **200-200** | **174-194** | **221-223** | **148-148** | **106-115** | **143-169** | **167-169** | **236-272** | **247-247** | **243-251** | **200-218** |
| Cape Town 12 | **200-200** | **176-180** | **211-223** | **148-149** | **106-106** | **139-181** | **167-171** | **228-256** | **247-247** | **233-243** | **213-215** |
| Cape Town 13 | **200-200** | **180-260** | **211-219** | **149-149** | **100-118** | **161-171** | **167-195** | **262-278** | **247-247** | **241-277** | **210-219** |
| Cape Town 14 | **200-202** | **164-188** | **219-237** | **146-149** | **106-106** | **125-141** | **167-173** | **214-238** | **247-247** | **241-253** | **212-213** |
| Cape Town 15 | **200-200** | **164-164** | **221-243** | **147-148** | **106-106** | **123-161** | **171-171** | **234-234** | **247-247** | **233-247** | **211-219** |
| Cape Town 16 | **200-200** | **188-192** | **221-223** | **148-149** | **106-106** | **135-173** | **181-193** | **214-222** | **247-247** | **233-233** | **211-212** |
| Cape Town 17 | **200-200** | **168-168** | **219-225** | **146-150** | **106-106** | **123-125** | **167-175** | **226-232** | **247-247** | **233-281** | **207-209** |
| Cape Town 18 | **200-200** | **166-166** | **217-217** | **149-151** | **106-106** | **123-163** | **167-195** | **226-246** | **247-247** | **233-247** | **206-215** |
| Cape Town 19 | **200-200** | **182-194** | **225-249** | **148-150** | **106-106** | **123-145** | **167-167** | **242-242** | **247-247** | **231-239** | **213-213** |
| Cape Town 20 | **202-202** | **170-198** | **213-251** | **148-148** | **106-106** | **165-169** | **175-175** | **214-244** | **247-247** | **231-239** | **206-213** |

* the invasive individuals assigned to both clusters C and E in the Structure analysis (i.e., the Camden Park population, Bringelly no 21, Mt Annan no 2, 8 & 17, and Lonsdale no 16 & 22) are indicated by an asterisk. When studying the nuclear SSR alleles of these individuals, hybrid profiles were always found on several loci. For example in NSW, individual no 2 from Mount Annan has a hybrid pattern at loci ssrOeUA-DCA01, ssrOeUA-DCA05, ssrOeUA-DCA09, ssrOeUA-DCA18 and PA(ATT)2 (alleles “*europaea*” in blue). In South Australia, Lonsdale individual no 22 has a hybrid pattern at loci ssrOeUA-DCA03, ssrOeUA-DCA05, ssrOeUA-DCA18, and EMO3 (alleles “*cuspidata*” in red).

**Table S5.** Pairwise genetic differentiation (*F_ST_*, in percent) between invasive populations based on nuclear DNA SSRs. For South Australian populations *F_ST_* based on chlorotypes are also given in brackets. Abbreviations: Sheph. Hill = Shepherds Hill; Brown. Creek = Brownhill Creek; Camd. Park = Camden Park; Lonsd. = Lonsdale; Ludden. = Luddenham; Maitl. Park = Maitland Park.

| Pop. | Sheph. Hill | | Brown. Creek | Lonsd. | Camd. Park | Mont Annan | Bringelly | Ludden. | Maitl. Park | Harpers Hill |
| --- | --- | --- | --- | --- | --- | --- | --- | --- | --- | --- |
| Brown. Creek | 1.25^ns^  (20.57)*** |  | |  |  |  |  |  |  |  |
| Lonsd. | 2.60**  (31.85)*** | 0.55 ^ns^  (39.37)*** | |  |  |  |  |  |  |  |
| Camd. Park | 13.91*** | 14.02*** | | 14.09*** |  |  |  |  |  |  |
| Mont Annan | 30.27*** | 30.43*** | | 29.08*** | 15.18*** |  |  |  |  |  |
| Bringelly | 30.63*** | 30.64*** | | 29.05*** | 16.16*** | 7.23*** |  |  |  |  |
| Ludden. | 33.46*** | 33.55*** | | 31.67*** | 16.76*** | 8.97*** | 3.25** |  |  |  |
| Maitl. Park | 28.96*** | 29.46*** | | 28.02*** | 19.58*** | 16.94** | 14.34** | 18.81*** |  |  |
| Harpers Hill | 31.31*** | 31.60*** | | 29.98*** | 16.26*** | 9.97*** | 9.06*** | 10.10*** | 4.72 ^ns^ |  |
| Maui | 39.42*** | 38.50*** | | 36.58*** | 28.77*** | 26.31*** | 20.40*** | 22.84*** | 27.55** | 27.71*** |

ns = non significant; ** *P* < 0.01; *** *P* < 0.001

Based on this analysis, populations from South Australia and NSW are clearly differentiated (*F_ST_* ranging from 28.02 to 33.46%; when Camden Park is excluded).

In SA, Brownhill Creek population was not significantly differentiated from Lonsdale and Shepherds Hill populations (*F_ST_* = 0.55 and 1.25%, respectively). On the other hand, the genetic differentiation between Lonsdale and Shepherds Hill was significant but remains relatively low (*F_ST_* = 2.60%). These results suggest that there are high levels of gene flow by pollen between South Australian invasive populations. In contrast, based on maternal markers (chlorotypes), levels of differentiation between these populations are high (ranging from 20.57 to 39.37%).

In NSW, most of populations were significantly differentiated from each other based on nuclear SSRs. The lower levels of differentiation were found between Bringelly and Luddenham (*F_ST_* = 3.25%) that are geographically very close (6-7 km), and between Maitland Park and Harpers Hill (*F_ST_* = 4.72%) in the Central Hunter region. Camden Park appears highly differentiated from other East Australian populations (*F_ST_* ranging from 15.18 to 16.26%), even from Mount Annan which is located at only 5‑6 km from this locality. Comparatively, Camden Park was not more differentiated from South Australian populations (*F_ST_* ranging from 13.91 to 14.09%), which are located at more than 1000 km. Note that the Maui population is highly differentiated from all NSW populations (*F_ST_* > 20%).

**Table S6**. Allele size range (in bp), number of alleles (*N_a_*), allelic richness (*R_S_* for 20 individuals), observed and expected heterozygosities (*H_O_* and *H_S_* respectively) for each SSR locus for the three invasive populations of subsp. *europaea*. Note that Lonsdale analyses were repeated without the two trees that were detected to be admixed (Figure 2), as such individuals would potentially increase the values of *N_a_*, *R_s_*, *H_o_* and *H_s_*. *F_IS_* values are not significantly different from 0 for all populations.

|  | **Lonsdale** | | | | | **Lonsdale without no 16 and 22** | | | | | **Shepherds Hill** | | | | | | **Brownhill Creek** | | | | |  |
| --- | --- | --- | --- | --- | --- | --- | --- | --- | --- | --- | --- | --- | --- | --- | --- | --- | --- | --- | --- | --- | --- | --- |
| **Locus** | **Allele size range** | ***N_a_*** | ***R_s_*** | ***H_o_*** | ***H_s_*** | **Allele size range** | ***N_a_*** | ***R_s_*** | ***H_o_*** | ***H_s_*** | | **Allele Size Range** | ***N_a_*** | ***R_s_*** | ***H_o_*** | ***H_s_*** | **Allele Size Range** | ***N_a_*** | ***R_s_*** | ***H_o_*** | ***H_s_*** | |
|  |  |  |  |  |  |  |  |  |  |  | |  |  |  |  |  |  |  |  |  |  | |
| **DCA1** | 208-278 | 6 | 5.56 | 0.73 | 0.73 | 208-278 | 6 | 5.64 | 0.75 | 0.74 | | 208-276 | 6 | 6.00 | 0.85 | 0.74 | 208-276 | 5 | 4.68 | 0.76 | 0.73 | |
| **DCA3** | 233-255 | 8 | 7.22 | 0.83 | 0.77 | 235-255 | 7 | 6.62 | 0.82 | 0.74 | | 235-251 | 5 | 5.00 | 0.70 | 0.78 | 235-251 | 5 | 5.00 | 0.83 | 0.74 | |
| **DCA5** | 195-209 | 8 | 7.59 | 0.80 | 0.80 | 195-209 | 8 | 7.40 | 0.79 | 0.78 | | 195-207 | 6 | 6.00 | 0.85 | 0.81 | 195-207 | 6 | 5.68 | 0.72 | 0.72 | |
| **DCA8** | 127-153 | 11 | 10.50 | 0.90 | 0.90 | 127-153 | 11 | 10.53 | 0.89 | 0.89 | | 127-149 | 8 | 8.00 | 0.70 | 0.86 | 127-153 | 10 | 9.05 | 0.90 | 0.86 | |
| **DCA9** | 163-213 | 13 | 11.28 | 0.90 | 0.86 | 163-213 | 13 | 11.52 | 0.89 | 0.86 | | 163-211 | 10 | 10.00 | 0.90 | 0.88 | 163-211 | 9 | 8.37 | 0.79 | 0.86 | |
| **DCA14** | 149-190 | 6 | 5.45 | 0.73 | 0.61 | 149-190 | 6 | 5.35 | 0.71 | 0.60 | | 157-190 | 8 | 8.00 | 0.80 | 0.75 | 173-190 | 5 | 4.99 | 0.72 | 0.72 | |
| **DCA15** | 247-271 | 4 | 4.00 | 0.73 | 0.67 | 247-271 | 4 | 4.00 | 0.71 | 0.68 | | 247-271 | 4 | 4.00 | 0.75 | 0.68 | 247-271 | 5 | 4.88 | 0.66 | 0.72 | |
| **DCA18** | 168-218 | 8 | 7.78 | 0.70 | 0.82 | 168-182 | 7 | 6.92 | 0.68 | 0.81 | | 168-184 | 8 | 8.00 | 0.90 | 0.87 | 168-182 | 7 | 6.99 | 0.76 | 0.85 | |
| **EMO3** | 213-225 | 7 | 6.87 | 0.80 | 0.82 | 218-225 | 6 | 5.99 | 0.79 | 0.81 | | 218-226 | 8 | 8.00 | 0.90 | 0.85 | 218-225 | 6 | 5.69 | 0.76 | 0.80 | |
| **GAPU71A** | 211-237 | 5 | 4.93 | 0.63 | 0.57 | 211-237 | 5 | 4.96 | 0.64 | 0.58 | | 211-235 | 5 | 5.00 | 0.75 | 0.72 | 211-233 | 5 | 4.38 | 0.52 | 0.55 | |
| **PA(ATT)2** | 106-124 | 6 | 5.66 | 0.70 | 0.78 | 106-124 | 6 | 5.71 | 0.71 | 0.78 | | 106-124 | 6 | 6.00 | 0.85 | 0.75 | 106-124 | 6 | 5.97 | 0.76 | 0.79 | |
|  |  |  |  |  |  |  |  |  |  |  | |  |  |  |  |  |  |  |  |  |  | |
| **Average^a^** | - | 7.8 | 7.28 | 0.77 | 0.77 | - | 7.5 | 7.06 | 0.77 | 0.76 | | - | 7.0 | 7.00 | 0.82 | 0.80 | - | 6.4 | 6.08 | 0.75 | 0.76 | |

**^a^** DCA15 was not considered to compute average values because not variable in subsp. *cuspidata*.

**Table S7.** Allele size range (in bp), number of alleles (*N_a_*), allelic richness (*R_S_* for 20 individuals), observed and expected heterozygosities (*H_O_* and *H_S_* respectively) for each SSR locus for the six invasive populations of subsp. *cuspidata*. Note that Bringelly and Mount Annan analyses were repeated without the trees detected as inter-subspecies hybrids (Figure 2), as such individuals would potentially increase the values of *N_a_*, *R_s_*, *H_o_* and *H_s_*. *F_IS_* values are not significantly different from 0, excepted for Camden Park and Maui that exhibits an excess of heterozygoty.

|  | **Camden Park** | | | | | | | **Mount Annan** | | | | | | | | | | | **Mount Annan without no 2, 8 and 17** | | | | | | | | | | | **Luddenham** | | | | | | | | | | |  |  |
| --- | --- | --- | --- | --- | --- | --- | --- | --- | --- | --- | --- | --- | --- | --- | --- | --- | --- | --- | --- | --- | --- | --- | --- | --- | --- | --- | --- | --- | --- | --- | --- | --- | --- | --- | --- | --- | --- | --- | --- | --- | --- | --- |
| **Locus** | **Allele size range** | ***N_a_*** | ***R_s_*** | ***H_o_*** | | ***H_s_*** | | | **Allele size range** | | ***N_a_*** | | ***R_s_*** | | ***H_o_*** | | ***H_s_*** | | | **Allele size range** | | ***N_a_*** | | ***R_s_*** | | ***H_o_*** | | ***H_s_*** | | | **Allele size range** | | ***N_a_*** | | ***R_s_*** | | ***H_o_*** | | ***H_s_*** | | |  |
|  |  |  |  |  | |  | | |  | |  | |  | |  | |  | | |  | |  | |  | |  | |  | | |  | |  | |  | |  | |  | | |  |
| **DCA1** | 208-276 | 6 | 5.96 | 0.88 | | 0.79 | | | 214-276 | | 8 | | 7.49 | | 0.72 | | 0.77 | | | 214-244 | | 6 | | 5.90 | | 0.68 | | 0.74 | | | 214-260 | | 6 | | 5.73 | | 0.76 | | 0.71 | | |  |
| **DCA3** | 233-257 | 4 | 3.99 | 0.72 | | 0.63 | | | 233-241 | | 3 | | 2.80 | | 0.52 | | 0.53 | | | 233-237 | | 2 | | 2.00 | | 0.50 | | 0.51 | | | 233-237 | | 2 | | 1.99 | | 0.12 | | 0.12 | | |  |
| **DCA5** | 199-207 | 5 | 4.76 | 0.72 | | 0.67 | | | 199-202 | | 4 | | 3.60 | | 0.32 | | 0.28 | | | 200-202 | | 2 | | 2.00 | | 0.27 | | 0.24 | | | 200-202 | | 2 | | 2.00 | | 0.28 | | 0.25 | | |  |
| **DCA8** | 125-145 | 6 | 5.79 | 0.64 | | 0.76 | | | 123-143 | | 6 | | 5.56 | | 0.56 | | 0.58 | | | 123-143 | | 4 | | 3.90 | | 0.55 | | 0.50 | | | 123-141 | | 5 | | 4.88 | | 0.36 | | 0.33 | | |  |
| **DCA9** | 167-219 | 9 | 8.36 | 0.88 | | 0.82 | | | 167-233 | | 11 | | 10.28 | | 0.92 | | 0.84 | | | 167-233 | | 10 | | 9.71 | | 0.91 | | 0.85 | | | 167-237 | | 11 | | 10.14 | | 0.72 | | 0.80 | | |  |
| **DCA14** | 148-190 | 6 | 5.76 | 0.80 | | 0.73 | | | 146-150 | | 5 | | 4.59 | | 0.72 | | 0.55 | | | 146-150 | | 5 | | 4.82 | | 0.73 | | 0.57 | | | 146-150 | | 4 | | 3.99 | | 0.68 | | 0.62 | | |  |
| **DCA15** | 247-271 | 4 | 3.96 | 0.56 | | 0.61 | | | 247-258 | | 2 | | 1.80 | | 0.04 | | 0.04 | | | 247 | | 1 | | 1.00 | | - | | - | | | 247 | | 1 | | 1.00 | | - | | - | | |  |
| **DCA18** | 168-220 | 9 | 8.56 | 1.00 | | 0.85 | | | 170-220 | | 8 | | 7.72 | | 0.64 | | 0.77 | | | 170-220 | | 7 | | 6.90 | | 0.64 | | 0.76 | | | 170-218 | | 7 | | 6.96 | | 0.76 | | 0.82 | | |  |
| **EMO3** | 212-225 | 6 | 5.80 | 0.88 | | 0.77 | | | 208-225 | | 5 | | 4.80 | | 0.68 | | 0.73 | | | 208-213 | | 4 | | 4.00 | | 0.64 | | 0.71 | | | 208-213 | | 4 | | 3.96 | | 0.56 | | 0.50 | | |  |
| **GAPU71A** | 211-237 | 6 | 5.80 | 0.88 | | 0.81 | | | 213-249 | | 8 | | 7.52 | | 0.60 | | 0.58 | | | 213-249 | | 8 | | 7.81 | | 0.64 | | 0.61 | | | 213-237 | | 5 | | 5.00 | | 0.80 | | 0.79 | | |  |
| **PA(ATT)2** | 106-124 | 6 | 5.59 | 0.56 | | 0.47 | | | 106-118 | | 4 | | 3.60 | | 0.44 | | 0.36 | | | 106-118 | | 3 | | 2.91 | | 0.41 | | 0.34 | | | 100-118 | | 4 | | 3.60 | | 0.64 | | 0.48 | | |  |
|  |  |  |  |  | |  | | |  | |  | |  | |  | |  | | |  | |  | |  | |  | |  | | |  | |  | |  | |  | |  | | |  |
| **Average^a^** |  | 6.3 | 6.04 | 0.80 | | 0.73 | | |  | | 6.2 | | 5.80 | | 0.61 | | 0.60 | | |  | | 5.1 | | 5.00 | | 0.60 | | 0.58 | | |  | | 5.0 | | 4.83 | | 0.57 | | 0.54 | | |  |
|  |  |  |  |  | |  | | |  | |  | |  | |  | |  | | |  | |  | |  | |  | |  | | |  | |  | |  | |  | |  | | |  |
|  | **Bringelly** | | | | | | | **Bringelly without individual no 21** | | | | | | | | | | | **Harpers Hill** | | | | | | | | | | | **Maui** | | | | | | | | | | |  |  |
| **Locus** | **Allele size range** | ***N_a_*** | ***R_s_*** | | ***H_o_*** | | ***H_s_*** | | | **Allele size range** | | ***N_a_*** | | ***R_s_*** | | ***H_o_*** | | ***H_s_*** | | | **Allele size range** | | ***N_a_*** | | ***R_s_*** | | ***H_o_*** | | ***H_s_*** | | | **Allele size range** | | ***N_a_*** | | ***R_s_*** | | ***H_o_*** | | ***H_s_*** | | |
|  |  |  |  | |  | |  | | |  | |  | |  | |  | |  | | |  | |  | |  | |  | |  | | |  | |  | |  | |  | |  | | |
| **DCA1** | 214-260 | 6 | 5.66 | | 0.79 | | 0.77 | | | 214-260 | | 6 | | 5.69 | | 0.82 | | 0.77 | | | 208-244 | | 8 | | 7.66 | | 0.83 | | 0.80 | | | 214-242 | | 3 | | 2.95 | | 0.54 | | 0.47 | | |
| **DCA3** | 233-253 | 3 | 2.69 | | 0.31 | | 0.43 | | | 233-237 | | 2 | | 2.00 | | 0.28 | | 0.42 | | | 233-237 | | 2 | | 1.98 | | 0.08 | | 0.08 | | | 233-237 | | 2 | | 2.00 | | 0.62 | | 0.48 | | |
| **DCA5** | 200-207 | 3 | 2.91 | | 0.38 | | 0.44 | | | 200-202 | | 2 | | 2.00 | | 0.39 | | 0.40 | | | 200-202 | | 2 | | 2.00 | | 0.38 | | 0.36 | | | 200-202 | | 2 | | 2.00 | | 0.15 | | 0.15 | | |
| **DCA8** | 123-143 | 6 | 4.67 | | 0.17 | | 0.20 | | | 123-143 | | 4 | | 3.35 | | 0.14 | | 0.14 | | | 123-141 | | 5 | | 4.98 | | 0.79 | | 0.68 | | | 125-133 | | 2 | | 2.00 | | 0.58 | | 0.42 | | |
| **DCA9** | 167-229 | 11 | 9.64 | | 0.86 | | 0.86 | | | 167-229 | | 10 | | 9.04 | | 0.86 | | 0.85 | | | 169-233 | | 5 | | 5.00 | | 0.79 | | 0.74 | | | 167-169 | | 2 | | 2.00 | | 0.42 | | 0.51 | | |
| **DCA14** | 146-150 | 6 | 5.38 | | 0.69 | | 0.73 | | | 146-150 | | 5 | | 4.71 | | 0.71 | | 0.72 | | | 146-150 | | 5 | | 4.88 | | 0.92 | | 0.74 | | | 146-149 | | 2 | | 2.00 | | 0.62 | | 0.43 | | |
| **DCA15** | 247 | 1 | 1.00 | | - | | - | | | 247 | | 1 | | 1.00 | | - | | - | | | 247 | | 1 | | 1.00 | | - | | - | | | 247 | | 1 | | 1.00 | | - | | - | | |
| **DCA18** | 164-218 | 12 | 10.25 | | 0.86 | | 0.82 | | | 164-218 | | 11 | | 9.69 | | 0.86 | | 0.81 | | | 164-218 | | 11 | | 10.16 | | 0.79 | | 0.83 | | | 170-186 | | 2 | | 2.00 | | 0.54 | | 0.51 | | |
| **EMO3** | 208-223 | 5 | 4.68 | | 0.66 | | 0.58 | | | 208-223 | | 4 | | 4.00 | | 0.68 | | 0.57 | | | 207-213 | | 4 | | 3.97 | | 0.54 | | 0.54 | | | 211-213 | | 3 | | 2.95 | | 0.38 | | 0.52 | | |
| **GAPU71A** | 211-249 | 9 | 7.74 | | 0.72 | | 0.71 | | | 213-249 | | 7 | | 6.42 | | 0.36 | | 0.69 | | | 213-249 | | 7 | | 6.67 | | 0.71 | | 0.81 | | | 213-249 | | 3 | | 2.77 | | 0.38 | | 0.35 | | |
| **PA(ATT)2** | 106-118 | 3 | 3.00 | | 0.55 | | 0.57 | | | 106-118 | | 3 | | 3.00 | | 0.57 | | 0.58 | | | 106-118 | | 2 | | 2.00 | | 0.25 | | 0.28 | | | 106-118 | | 2 | | 2.00 | | 0.50 | | 0.42 | | |
|  |  |  |  | |  | |  | | |  | |  | |  | |  | |  | | |  | |  | |  | |  | |  | | |  | |  | |  | |  | |  | | |
| **Average^a^** |  | 6.4 | 5.66 | | 0.60 | | 0.61 | | |  | | 5.4 | | 4.99 | | 0.57 | | 0.60 | | |  | | 5.1 | | 4.93 | | 0.61 | | 0.59 | | |  | | 2.3 | | 2.27 | | 0.47 | | 0.43 | | |
|  |  |  |  | |  | |  | | |  | |  | |  | |  | |  | | |  | |  | |  | |  | |  | | |  | |  | |  | |  | |  | | |

**^a^** DCA15 was not considered to compute average values because not variable in subsp. *cuspidata*; ^b^ At Camden Park, the *F_IS_* value was negative and significantly different from 0 (*F_IS_* = ‑0.078; CI_95%_ = [-0.178 ‒ -0.026]); ^c^ At Maui, the *F_IS_* value was negative and significantly different from 0 (*F_IS_* = ‑0.116; CI_95%_ = [‑0.230 ‒ -0.044]).

**Table S8.** Type-1 and type-2 error rates for simulated data. This table shows the results of our ABC algorithm when data are simulated under one model (“true scenario”). The algorithm identifies the most probable scenario for each of 300 independent data sets (one hundred per scenario) using the logistic regression approach. The error rates are computed using the “confidence in model choice” function, implemented in DiyABC (see Materials and Methods for details).

| True |  | Type-2 error | | |  | Type-1 |
| --- | --- | --- | --- | --- | --- | --- |
| scenario |  | **1** | **2** | **3** |  | **error** |
| 1 |  | - | 0.100 | 0.020 |  | 0.142 |
| 2 |  | 0.136 | - | 0.010 |  | 0.138 |
| 3 |  | 0.036 | 0.038 | - |  | 0.037 |

**Table S9.** Precision on parameter estimations based on 500 pseudo-observed data sets (pods) with the measure of the relative average bias, estimated under scenario 1. The pods are drawn from the prior distribution given in Table 1

| **Parameter** | **Relative average bias** | | |
| --- | --- | --- | --- |
|  | **Mean** | **Median** | **Mode** |
| N_e1_ | 0.213 | 0.149 | -0.005 |
| N_e2_ | 0.350 | 0.352 | 0.343 |
| N_e3_ | 0.378 | 0.379 | 0.391 |
| N_1_ | 0.125 | 0.112 | 0.137 |
| N_2_ | 0.256 | 0.236 | 0.28 |
| db_1_ | 0.063 | 0.06 | 0.082 |
| db_2_ | 0.152 | 0.076 | -0.130 |
| T_1_ | 0.003 | 0.034 | 0.169 |
| T_2_ | 0.073 | 0.057 | 0.013 |
| mu | 0.143 | 0.068 | -0.126 |

**Table S10.** Precision on parameter estimations based on 500 pseudo-observed data sets (pods) with the measure of the square Root of the Relative Mean Square Error (RRMSE) and the Relative Median Absolute Deviation (RMedAD), estimated under scenario 1. The pods are drawn from the prior distribution given in Table 1.

| **Parameter** | **RRMSE** | | | **RMedAD** |
| --- | --- | --- | --- | --- |
|  | **Mean** | **Median** | **Mode** |  |
| N_e1_ | 0.551 | 0.488 | 0.430 | 0.349 |
| N_e2_ | 1.017 | 1.022 | 1.400 | 0.434 |
| N_e3_ | 1.089 | 1.088 | 1.383 | 0.430 |
| N_1_ | 0.720 | 0.721 | 0.838 | 0.337 |
| N_2_ | 0.910 | 0.876 | 0.974 | 0.386 |
| db_1_ | 0.537 | 0.541 | 0.582 | 0.293 |
| db_2_ | 0.925 | 0.835 | 0.746 | 0.427 |
| T_1_ | 0.264 | 0.275 | 0.368 | 0.147 |
| T_2_ | 0.514 | 0.487 | 0.440 | 0.270 |
| mu | 0.553 | 0.481 | 0.448 | 0.368 |

**Table S11.** Model checking for introduction scenario 1. The probability for each test quantity (t) was computed from 1,000 data sets simulated from the posterior distribution of parameters obtained under scenario 1. The test quantities correspond to the summary statistics used to discriminate among scenarios and compute the posterior distribution of parameters and some supplementary statistics. Corresponding tail-area probabilities, or p-values, of the test quantities (t) can be easily obtained as Prob (t_simulated_ < t_observed_) and 1.0 - Prob (t_simulated_ < t_observed_) for Prob (t_simulated_ < t_observed_) ≤ 0.5 and > 0.5, respectively. NAL _i_ = mean number of alleles in the population i, HET _i_ = mean expected heterozygosity in the population i, VAR _i_ = mean allelic size variance in the population i, MGW _i_ = mean ratio of the number of alleles over the range of allele sizes in the population i, *F_ST_*_( i/j)_ = *F_ST_* value between population i and j, N2P _(i & j)_ = mean number of alleles in the pooled populations i and j, H2P _(i & j)_ = mean expected heterozygosity pooling samples from populations i and j, V2P _(i & j)_ = mean allelic size variance pooling samples from population i and j. Population abbreviations: SA = South Africa, NSW = New South Wales.

| **Test quantity (t)** | **Observed Value** | ***p*-value** |
| --- | --- | --- |
| NAL _SA_ | 11.91 | 0.532 |
| HET _SA_ | 0.72 | 0.006 (**) |
| VAR _SA_ | 38.76 | 0.709 |
| MGW _SA_ | 0.57 | 0.094 |
|  |  |  |
| NAL _NSW_ | 7.09 | 0.264 |
| HET _NSW_ | 0.56 | 0.008 (**) |
| VAR _NSW_ | 28.68 | 0.599 |
| MGW _NSW_ | 0.54 | 0.444 |
|  |  |  |
| NAL _Hawaii_ | 2.18 | 0.273 |
| HET _Hawaii_ | 0.39 | 0.381 |
| VAR _Hawaii_ | 10.30 | 0.455 |
| MGW _Hawaii_ | 0.35 | 0.462 |
|  |  |  |
| *F_ST_* _(SA/NSW)_ | 0.14 | 0.968 (*) |
| *F_ST_* _(SA/Hawaii)_ | 0.28 | 0.567 |
| *F_ST_* _(NSW/Hawaii)_ | 0.19 | 0.508 |
|  |  |  |
| N2P _(SA & NSW)_ | 14.18 | 0.612 |
| N2P (_SA & Hawaii)_ | 12.09 | 0.475 |
| N2P _(NSW & Hawaii)_ | 7.09 | 0.209 |
|  |  |  |
| H2P _(SA & NSW)_ | 0.61 | 0.003 (**) |
| H2P _(SA & Hawaii)_ | 0.63 | 0.050 (*) |
| H2P _(NSW & Hawaii)_ | 0.58 | 0.006 (**) |
|  |  |  |
| V2P _(SA & NSW)_ | 32.41 | 0.684 |
| V2P _(SA & Hawaii)_ | 26.76 | 0.591 |
| V2P _(NSW & Hawaii)_ | 29.04 | 0.622 |

(*), (**): tail-area probability < 0.05 and < 0.01, respectively

**Table S12.** Effect of different priors on the posteriors for the real data sets. This table shows the robustness of our inference to prior choice for parameters estimates. All the results presented here were obtained under scenario 1 using the DiyABC software as applied to the olive tree data. Prior set 1: this is the standard set of priors described in Table 1 and in the main text; prior set 2: differs from prior set 1 only by the fact that we assume the Generalized Stepwise Mutation model (GSM) instead of the SMM; prior set 3: differs from prior set 1 only by the fact that we assume logUniform distributions for N_e1_, N_e2_, N_e3_, N_1_ and N_2_ while keeping the same range; prior set 4: differs from prior set 1 by the fact that the db_1_ and db_2_ priors are much narrower (we use U[1, 10] instead of U[1, 40]).

| Parameter | Prior set | Mean | Median | Mode | Q_2.5%_ | Q_97.5%_ |
| --- | --- | --- | --- | --- | --- | --- |
| N_e1_ | 1 | 33ʹ500 | 32ʹ000 | 16ʹ000 | 11ʹ200 | 64ʹ800 |
|  | 2 | 13ʹ200 | 12ʹ500 | 10ʹ100 | 10ʹ100 | 20ʹ700 |
|  | 3 | 26ʹ900 | 24ʹ400 | 11ʹ000 | 10ʹ600 | 55ʹ800 |
|  | 4 | 27ʹ100 | 24ʹ800 | 10ʹ400 | 10ʹ600 | 54ʹ700 |
| N_e2_ | 1 | 51ʹ800 | 49ʹ700 | 21ʹ600 | 11ʹ600 | 97ʹ200 |
|  | 2 | 46ʹ400 | 42ʹ200 | 11ʹ000 | 11ʹ100 | 96ʹ000 |
|  | 3 | 38ʹ500 | 30ʹ800 | 10ʹ400 | 10ʹ500 | 88ʹ300 |
|  | 4 | 41ʹ200 | 34ʹ900 | 12ʹ500 | 10ʹ900 | 94ʹ300 |
| N_e3_ | 1 | 62ʹ300 | 65ʹ700 | 98ʹ200 | 13ʹ600 | 98ʹ500 |
|  | 2 | 54ʹ800 | 55ʹ000 | 57ʹ300 | 12ʹ300 | 97ʹ600 |
|  | 3 | 30ʹ700 | 22ʹ500 | 10ʹ100 | 10ʹ200 | 88ʹ300 |
|  | 4 | 54ʹ800 | 54ʹ500 | 20ʹ900 | 12ʹ300 | 97ʹ900 |
| N_1_ | 1 | 27.5 | 27.5 | 25.6 | 6.65 | 48.3 |
|  | 2 | 25.1 | 24.6 | 28.1 | 5.7 | 48 |
|  | 3 | 28.4 | 28.7 | 32.3 | 7.3 | 48.8 |
|  | 4 | 30.8 | 32.1 | 34.8 | 9.8 | 47.4 |
| N_2_ | 1 | 16.7 | 14.3 | 9.2 | 2.99 | 44 |
|  | 2 | 14.6 | 14.2 | 5.9 | 2.6 | 42.5 |
|  | 3 | 15.1 | 13.2 | 5.5 | 2.9 | 40.5 |
|  | 4 | 5.8 | 4.5 | 3.4 | 1.9 | 20.1 |
| db_1_ | 1 | 16.7 | 14.3 | 9.2 | 2.99 | 44 |
|  | 2 | 5.1 | 4.47 | 2 | 1 | 13.5 |
|  | 3 | 6.3 | 5.6 | 4.5 | 1.2 | 15.9 |
|  | 4 | 7.7 | 8.3 | 10 | 2.1 | 10 |
| db_2_ | 1 | 12.2 | 11.6 | 11.5 | 1.85 | 27.7 |
|  | 2 | 14.6 | 14.2 | 13.7 | 2.39 | 29.1 |
|  | 3 | 9.6 | 8.7 | 4.1 | 1.6 | 22.9 |
|  | 4 | 5.9 | 6 | 6 | 1.2 | 9.8 |
| K_1_ | 1 | 3.6 | 3.3 | 3.1 | 1.5 | 6.7 |
|  | 2 | 4.2 | 3.9 | 3.6 | 2.0 | 8.1 |
|  | 3 | 2.7 | 2.4 | 2.1 | 1.5 | 5.5 |
|  | 4 | 4.0 | 3.5 | 2.9 | 1.8 | 10.1 |
| K_2_ | 1 | 1.0 | 0.8 | 0.6 | 0.3 | 2.5 |
|  | 2 | 1.1 | 0.8 | 0.7 | 0.4 | 3.2 |
|  | 3 | 1.0 | 1.0 | 0.8 | 0.5 | 2.1 |
|  | 4 | 1.0 | 0.9 | 0.8 | 0.5 | 2.1 |
| T_1_ | 1 | 35.1 | 36.4 | 38 | 23.2 | 40 |
|  | 2 | 32.3 | 33.7 | 38 | 17.2 | 40 |
|  | 3 | 33.2 | 34.6 | 38 | 19.1 | 40 |
|  | 4 | 34.6 | 35.9 | 38 | 22.4 | 40 |
| T_2_ | 1 | 19.2 | 19 | 18 | 6.61 | 33.1 |
|  | 2 | 17.8 | 17.3 | 15.3 | 5.52 | 32.8 |
|  | 3 | 18 | 17.5 | 14.7 | 5.83 | 32.1 |
|  | 4 | 21.6 | 21.5 | 21.5 | 7.33 | 35.7 |
| mu | 1 | 0.00030 | 0.00028 | 0.00010 | 0.00010 | 0.00063 |
|  | 2 | 0.00014 | 0.00013 | 0.00010 | 0.00010 | 0.00025 |
|  | 3 | 0.00068 | 0.00076 | 0.00086 | 0.00012 | 0.00094 |
|  | 4 | 0.00029 | 0.00028 | 0.00010 | 0.00010 | 0.00061 |

Abbreviations: N_e1_, N_e2_ and N_e3_ = population effective sizes for South African, NSW and Hawaiian populations; N_1_ and N_2_ = number of funders for the first and second colonization events (in NSW and then Hawaii); db_1_ and db_2_ = duration of the initial bottleneck after introduction in NSW and Hawaii (number of generations); K_1_ and K_2_ = intensity of the bottleneck in NSW and Hawaii; T_1_ and T_2_ = introduction times for the two colonization events (number of generations); mu = SSR mutation rate.

**Figure S1.** The three demographic scenarios of African olive invasion in Australia and Hawaii

The parameters of the models are as follows; T_i_: invasion time of the i^th^ population; db_i_: duration of the bottleneck (or latency phase) for the i^th^ population; Ne_i_: current effective size for the i^th^ population. Each vertical box represents a population and the connections between each box symbolize introduction events. Note that in scenario 3, the order of introductions is chosen randomly based on the T_i_ values.

**
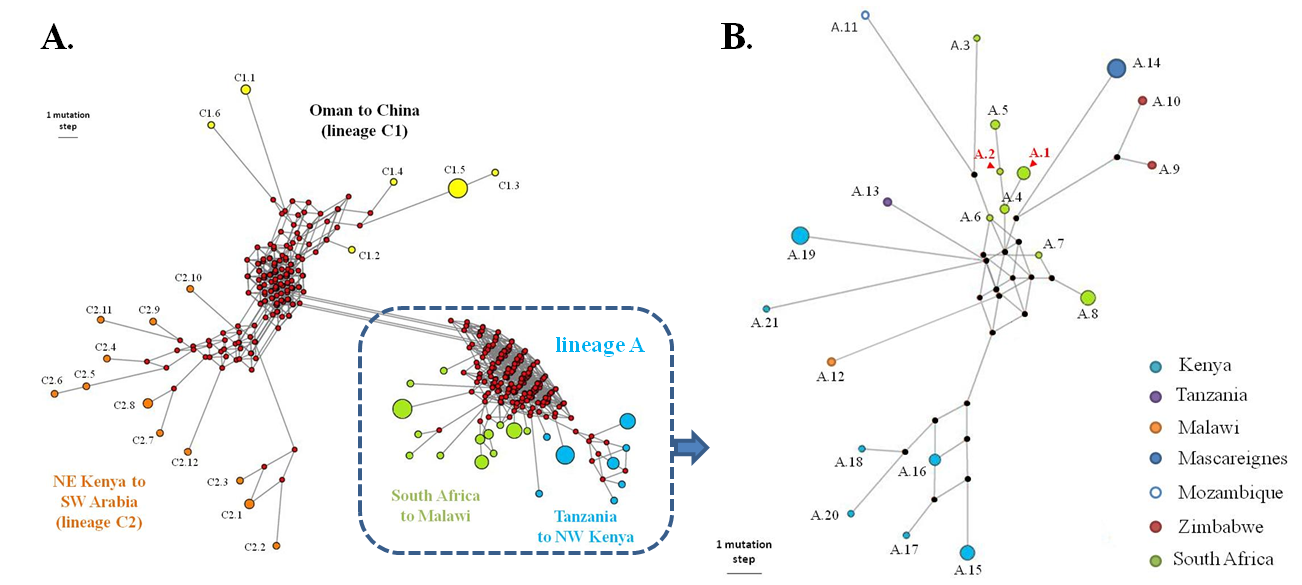
**

**Figure S2.** Reduced-median networks (Bandelt et al. 1999) of chlorotypes detected in the native range of subsp. *cuspidata*. Each chlorotype is represented by a dot, whose the width is proportional to the number of occurrences in our sample. See Table S3 for chlorotype profiles and geographic origins. The length of branches is proportional to the number of mutational steps. The missing, intermediate nodes are indicated by small red points. Lineages are named according to Besnard et al. (2007b).

**A -** Network of the 39 chlorotypes belonging to lineages A, C1 (yellow dots) and C2 (orange dots). Chlorotypes from southern Africa (South Africa to Mozambique) are represented in green whereas chlorotypes detected in central Africa (Malawi to NW Kenya) are in blue. The reticulation of network indicates that relationships between lineages are not clearly resolved. This is due to a high homoplasy in microsatellites when comparing too diverging lineages; **B ‑** Network of the 21 chlorotypes belonging to lineage A (Sub-Saharan olive). For each chlorotype, the country of origin is indicated by a specific color. The two African chlorotypes detected in NSW are indicated in red (A.1 and A.2; both were detected in the population of Cape Town, South Africa). The homoplasy is considerably reduced at the lineage level (compared to network S2A).

**Figure S3.** Barplot of the Structure analysis for the Australian and Hawaiian invasive olive populations based on the most probable number of genetic clusters *K* = 2 (according to criteria defined by Evanno et al. 2005). The analysis was performed based on eleven nuclear SSRs using Bayesian simulations. The percentage of assignment of each individual to the two clusters averaged over 10 iterations is shown. Each vertical bar represents an individual. Individuals identified as admixed individuals - i.e. early generations of hybrids - are indicated at the bottom of the figure (except for Camden Park where all individuals area admixed). The Mediterranean and African chloroplast lineages are indicated for all trees (in NSW, note that Mt Annan no 17 and Bringelly no 21 exhibit Mediterranean chlorotypes). The chloroplast lineages match with the two clusters defined on nuclear SSRs except for admixed individuals.

**
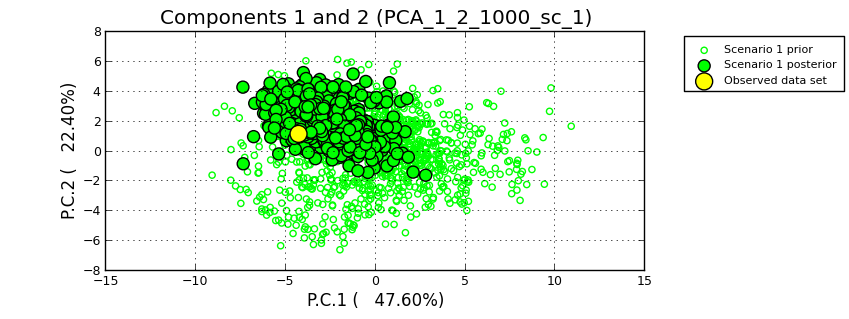
**

**Figure S4.** Principal Component Analysis (PCA) of the model checking computation in the space of summary statistics. The small green dots, the great green dots and the yellow dot respectively represent the prior distribution of parameters, the data set from the posterior predictive distribution, and the observed data.

**Supplemental Methods.** Alternative ABC implementation using in-house scripts, the ms program and the abc R package (R development core team 2010)

Different approaches can be applied to perform an ABC analysis. In this project, two approaches were used. Our principal analysis was performed with the DiyABC v.2.0 program (Cornuet et al. submitted), a user-friendly software which permits to perform easily ABC inferences (see main text). However, the DiyABC software has some limitations (admixture is allowed but gene flow cannot be simulated, population size change is possible but must be sudden, and cannot be linear or exponential). We therefore decided to additionally use an alternative approach which would serve two purposes. First it would allow us to validate our DiyABC inferences and test its robustness to some modeling assumption. Second it could serve as a first step towards building more complex models. We turned to a more flexible strategy whereby all the ABC steps would be carried out independently using different tools, namely in-house scripts and R packages.

Priors were simulated with the R software v. 2.14.1. The population genetic simulation step was achieved with ms (Hudson 2002), a software that generates genetic data under a very wide variety of neutral models. The ms program typically produces sequence data under the Infinite-Sites Mutation (ISM) model but can be coupled with the Microsat program (Minch et al. 1995) which transforms the ms output in microsatellite data assuming a simple Stepwise Mutation Model (SMM).

The computation of summary statistics was done using functions implemented in SPAms (Parreira et al. 2009), a user-friendly program that allows simulating genetic data under different demographic models. The functions originally written in C or MATLAB (Mathworks) were translated in the R programming language.

The final estimation procedure (rejection with or without regression) was performed with the abc R package (Csilléry et al. 2012). For comparison with the DiyABC approach, we used a logistic regression procedure to identify the most probable model and the linear regression procedure to estimate the demographic parameters.

Overall, the results obtained using the alternative ABC implementation provided the same results as the DiyABC approach. We also found that scenario 1 was the most strongly supported scenario with a posterior probability close to one, suggesting a sequential colonization with a first introduction to Australia from South Africa followed by a second introduction to Hawaii from Australia.

Summaries of the priors and posteriors are reported in Table SM1 and provide the same general trend as with DiyABC. However, there are some differences. For instance, the mode for the number of founders in Australia, N_1_, is close to 14 individuals, compared to 26 using DiyABC. The two values are on the same order but differ somewhat. The mode for the number of founders in Hawaii, N_2_, is 9 individuals under both analyses. The split parameters T_1_ and T_2_ also exhibit similar values under both approaches. Some differences appear with the latency phase parameters (db_1_ and db_2_) and the current effective size parameters (Ne_1_, Ne_2_ and Ne_3_).

Globally, all analyses performed with ms or DiyABC using different prior sets or demographic models gave parameter estimations that were consistent for the number of founders and for the timing of the introduction. Three main conclusions appear therefore to be robust: (*i*) the most supported scenario is clearly the scenario 1 (a first introduction from South Africa into Australia, followed by an introduction into Hawaii from Australia), and (*ii*) a reduced number of individuals (less than thirty individuals, and perhaps less than 20) was introduced into Australia and even fewer to Hawaii, and finally (*iii*) we estimated that between 35 and 40 generations probably have occurred since the first introduction in NSW.

**Table SM1.** Summary of the posterior distributions. The mean, median, mode and 0.025 and 0.975 quantiles are given for the demographic parameters and the mutation rate under scenario 1 with the alternative ABC approach described above. Abbreviations: N_e1_, N_e2_ and N_e3_ = population effective sizes for South African, NSW and Hawaiian populations; N_1_ and N_2_ = number of funders for the first and second colonization events (in NSW and then Hawaii); db_1_ and db_2_ = duration of the initial bottleneck after introduction in NSW and Hawaii (number of generations); T_1_ and T_2_ = introduction times for the two colonization events (number of generations); mu = SSR mutation rate.

|  |  | Mean | Median | Mode | Q2.5% | Q97.5% |
| --- | --- | --- | --- | --- | --- | --- |
| N_e1_ | Prior | 54979 | 54982 |  | 12246 | 97768 |
|  | Posterior | 24343 | 22883 | 21768 | 11478 | 47548 |
| N_e2_ | Prior | 55001 | 55036 |  | 12232 | 97744 |
|  | Posterior | 64381 | 66629 | 71522 | 21403 | 95551 |
| N_e3_ | Prior | 54974 | 54942 |  | 12243 | 97719 |
|  | Posterior | 30785 | 25446 | 15655 | 10855 | 81452 |
| N_1_ | Prior | 26 | 26 |  | 3.2 | 48.8 |
|  | Posterior | 17.8 | 16.6 | 13.6 | 6.4 | 35 |
| N_2_ | Prior | 26 | 26 |  | 3.2 | 48.8 |
|  | Posterior | 20.7 | 18.6 | 8.7 | 3.4 | 46.4 |
| db_1_ | Prior | 7.8 | 6.20 | 1.9 | 0.2 | 23.5 |
|  | Posterior | 10.8 | 8.1 | 1.8 | 1 | 33.1 |
| db_2_ | Prior | 7.8 | 6.2 | 1 | 0.2 | 23.5 |
|  | Posterior | 14.5 | 12.8 | 2.1 | 1 | 36 |
| T_1_ | Prior | 32.2 | 33.8 | 40 | 16.5 | 39.8 |
|  | Posterior | 34.9 | 38.6 | 39.6 | 24.7 | 40 |
| T_2_ | Prior | 16.6 | 16 | 8.3 | 3.6 | 32.4 |
|  | Posterior | 19.2 | 19.3 | 19 | 12.4 | 25.3 |
| mu | Prior | 0.00055 | 0.00055 |  | 0.00012 | 0.00098 |
|  | Posterior | 0.00030 | 0.00030 | 0.00030 | 0.00010 | 0.00060 |

**References**

Bandelt, H.J., P. Forster, and A. Röhl 1999. Median-joining networks for inferring intraspecific phylogenies. Molecular Biology and Evolution **16:**37–48.

Besnard, G., P. Henry, L. Wille, D. Cooke, and E. Chapuis 2007a. On the origin of the invasive olives (*Olea europaea* L., Oleaceae). Heredity **99:**608–619.

Besnard, G., P. Hernández , B. Khadari, G. Dorado, and V. Savolainen 2011. Genomic profiling of plastid DNA variation in the Mediterranean olive tree. BMC Plant Biology **11:**80.

Besnard, G., B. Khadari, M. Navascués, et al. 2013. The complex historyMazuecos-Fernandez M, El Bakkali A, Arrigo N, Baali-Cherif D, Brunini-Bronzini de Caraffa V, Santoni S, Vargas P, Savolainen V. The complex history of the olive tree: from Late Quaternary diversification of Mediterranean lineage to primary domestication in the northern Levant. Proceedings of the Royal Society, Series B **280:**20122833.

Besnard, G., R. Rubio de Casas, and P. Vargas 2007b. Plastid and nuclear DNA polymorphism reveals historical processes of isolation and reticulation in the olive tree complex (*Olea europaea*). Journal of Biogeography **34:**736–752.

Cornuet, J.M., P. Pudlo, J. Veyssier, A. Dehne-Garcia, M. Gautier, R. Leblois, J.M. Marin, and A. Estoup Submitted. DIYABC v2.0: a software to make Approximate Bayesian Computation inferences about population history using Single Nucleotide Polymorphism, DNA sequences and microsatellite data. Bioinformatics.

Csilléry, K., O. Francois, and M.G.B. Blum 2012. abc: an R package for Approximate Bayesian Computation (ABC). Methods in Ecology and Evolution **3**:475–479.

Evanno, G., S. Regnault, and J. Goudet 2005. Detecting the number of clusters of individuals using the software Structure: a simulation study. Molecular Ecology **14**:2611–2620.

Hudson R.R. 2002. Generating samples under a Wright-Fisher neutral model of genetic variation. Bioinformatics **18**:337–338.

Minch, E., A. Ruiz-Linares, D.B. Godstein, M.W. Feldmann, and L.L. Cavalli-Sforza 1995. Microsat (version 1.4d): a computer program for calculating various statistics on microsatellite allele data. Stanford University Medical Center, Stanford.

Parreira, B., M. Trussart, V. Sousa, R. Hudson, and L. Chikhi 2009. SPAms: A user-friendly software to simulate population genetics data under complex demographic models. Molecular Ecology Resources **9**:749–753.

R Development Core Team 2010. R: A language and environment for statistical computing. R Foundation for Statistical Computing, Vienna, Austria.
